# Monitoring of benthic eukaryotic communities in two tropical coastal lagoons through eDNA metabarcoding: a spatial and temporal approximation

**DOI:** 10.1038/s41598-022-13653-9

**Published:** 2022-06-16

**Authors:** Margoth L. Castro-Cubillos, Joe D. Taylor, Alicia Mastretta-Yanes, Francisco Benítez-Villalobos, Valentina Islas-Villanueva

**Affiliations:** 1 Programa de Doctorado en Ecología Marina, División de Estudios de Posgrado, Universidad del Mar Campus Puerto Ángel, Cd. Universitaria s/n, 70902 Oaxaca, Mexico; 2 UK Centre for Ecology & Hydrology, Maclean Building, Benson Lane, Crowmarsh Gifford, Wallingford, OX10 8BB UK; 3 Comisión Nacional para el Conocimiento y Uso de la Biodiversidad (CONABIO), Liga Periferico Insurgentes Sur 4903, 14010 Mexico city, Mexico; 4 Consejo Nacional de Ciencia y Tecnología (CONACYT), Av. de los Insurgentes Sur 1582, 03940 Mexico, Mexico; 5 Instituto de Recursos, Universidad del Mar Campus Puerto Ángel, Cd. Universitaria s/n, 70902 Oaxaca, Mexico; 6 Instituto de Genética, Universidad del Mar Campus Puerto Ángel, Cd. Universitaria s/n, 70902 Oaxaca, Mexico

**Keywords:** Biodiversity, Community ecology, Ecology, Biodiversity, Community ecology, Molecular ecology, Tropical ecology, Wetlands ecology

## Abstract

Tropical coastal lagoons are important ecosystems that support high levels of biodiversity and provide several goods and services. Monitoring of benthic biodiversity and detection of harmful or invasive species is crucial, particularly in relation to seasonal and spatial variation of environmental conditions. In this study, eDNA metabarcoding was used in two tropical coastal lagoons, Chacahua (CH) and Corralero (C) (Southern Mexican Pacific), to describe the benthic biodiversity and its spatial–temporal dynamics. The distribution of benthic diversity within the lagoons showed a very particular pattern evidencing a transition from freshwater to seawater. Although the two lagoon systems are similar in terms of the species composition of metazoans and microeukaryotes, our findings indicate that they are different in taxa richness and structure, resulting in regional partitioning of the diversity with salinity as the driving factor of community composition in CH. Harmful, invasive, non-indigenous species, bioindicators and species of commercial importance were detected, demonstrating the reach of this technique for biodiversity monitoring along with the continued efforts of building species reference libraries.

## Introduction

Coastal lagoons are among the most important ecosystems in the world. They function as ecotones between terrestrial, freshwater, intertidal and marine systems, and are highly productive. The value of the goods and services provided by coastal lagoons is among the highest of all natural systems due to nutrient recycling, direct harvesting, recreation and aesthetic value^1^. Coastal lagoons include different types of habitats such as mangroves, marshes and seagrass beds^2^. These habitats function as spawning areas, breeding areas, feeding zones and migration corridors for both vertebrates and invertebrates^3,4^. Despite its clear importance for ecosystems and services, lagoon biodiversity is not well characterised, particularly in tropical regions.

The high spatial–temporal environmental heterogeneity of lagoons suggests a broad spectrum of species may coexist^5,6^. Because of the relatively low water discharge rate of lagoons, they are favourable habitats for primary producers, which in turn favour secondary production^7,8^. Due to their transitional nature they have highly variable gradients of abiotic factors, such as temperature, salinity and oxygen, and high biological productivity^1^. In a large majority of coastal lagoons and brackish water systems, salinity is one of the most important environmental factors that determines the structure of biodiversity at local and regional levels^6,9–11^. Salinity may be similar or superior to that of seawater in some of these lagoons, depending largely on their hydrological characteristics, which are determined by their configuration, the entry of seawater (tidal range), precipitation and fresh water from rivers; and evaporation^12,13^.

Basic biodiversity data is lacking and little is known about the impact of spatial and temporal variation on communities within highly heterogeneous lagoon systems, especially regarding the benthos. This is particularly true for the smallest representatives of eukaryotic biodiversity. In particular, eukaryotic organisms have been shown to be effective indicators of pollution and anthropogenic disturbance^14,15^. Many such organisms have not been studied or are unidentifiable based on morphology alone, especially meiofauna and microeukaryotes^16^. Molecular techniques represent an ideal means of rapid identification and profiling of eukaryotic communities, as they provide several advantages over traditional characterization methods^17–19^.

For more efficient biodiversity monitoring in different ecosystems, a multi-taxon approach is necessary, along with the use of tools that allow large-scale monitoring^20^. The metabarcoding of environmental DNA (eDNA) allows researchers to obtain a large amount of data for monitoring and characterization studies and reveals the community spatial–temporal structure and composition in different systems^21–23^. In lagoons and estuaries investigations have focused on seasonal and temporal variability in eukaryotic plankton^24–26^, benthic microbial eukaryote^27,28^ and bacterial communities^29,30^; however, few studies have been carried out in tropical regions.

In this study we focus on two tropical lagoon systems: the Chacahua Pastoria (CH) and Corralero- Alotengo (C) systems, located in the Eastern Tropical Pacific^31^, in the Southern Mexican Pacific^32^. The former lies within the Lagunas de Chacahua National Park, is a natural protected area^33^, and has been classified as a RAMSAR site (Ramsar Convention on Wetlands of International Importance Especially as Waterfowl Habitat) since 2008 (rsis.ramsar.org), while the latter is not, and is periodically subjected to dredging for artisanal fisheries. Due to their geographical proximity and similar characteristics, it is expected that the structure and composition of the eukaryotic diversity would be similar. The Chacahua-Pastoria (CH) system is composed of two lagoons. It used to have a permanent connection with the sea through a natural entrance in Chacahua and an artificial one in Pastoria. The former was naturally closed in 1981 but it was reopened again in 1997 by Hurricane Paulina. Since 2003, Chacahua has been the only connection to the sea^34,35^. The Corrralero-Alotengo system (C) has a seasonal closing and opening cycle of the connection to the sea, regulated by freshwater inputs from land and coastal processes. The reduction of these inputs contributes to the clogging in the mouth and interior channels, which hinders the entry of seawater, nutrients and species^36^. Over the last few decades, both lagoon systems have shown increased sedimentation, due to the closure of their connection to the sea and the reduction of freshwater inputs coming from land. However, both represent great commercial and ecological relevance, since they sustain species of commercial importance such as pen shell (*Atrina maura*), white shrimp (*Litopenaeus vannamei*), brown shrimp (*Farfantepenaeus californiensis*), crystal shrimp (*Farfantepenaeus brevirostris*), blue shrimp (*Litopenaeus stylirostris*), Charru mussel (*Mytella charruana*) and others^37–39^; and they are potential laboratories for ecological research and bioprospecting. However, due to the increase in contaminating waste from the surrounding human populations, some parasitic or pathogenic species could become a source of diseases not only for the species that inhabit the lagoon, but also for humans.

In the present study, the spatial and seasonal (biannual) changes in benthic eukaryotic community composition were evaluated in two lagoon systems with varying levels of protection. The diversity of metazoans (macroeukaryotes and meioeukaryotes) and microeukaryotes was assessed separately by amplifying fragments of specific genes; Cytocrome Oxidase subunit I (COI) was used for metazoans and 18S rRNA (V4 region) for microeukaryotes, due to the taxonomic bias previously found in certain groups and the status of these markers as standardised barcodes for metazoans and microeukaryotes^40^. The use of eDNA Metabarcoding allowed us to describe the baseline biodiversity in order to develop an efficient way of monitoring these systems and to evaluate the effects of anthropogenic disturbances and their conservation status. Sediments were selected because, in aquatic systems, a greater number of MOTUs (Molecular Operational Taxonomic Units) are obtained in sediment samples than in water samples. However, this depends on the group of organisms of interest for each study^41^. The benthic community of these lagoons is the focus of the current study. The expected findings include: (1) clear differences in the composition of the metazoan and microeukaryote community between seasons, between lagoons and sections of the lagoon; (2) differences in the composition of the metazoan and microeukaryote community will be given by the environmental parameters; (3) a higher species richness compared to previous studies in these lagoons; (4) the detection of invasive or exotic species; and (5) higher levels of biodiversity in the lagoon system that experiences less anthropogenic influence.

## Materials and methods

### Study area and sampling

**Figure 1:**
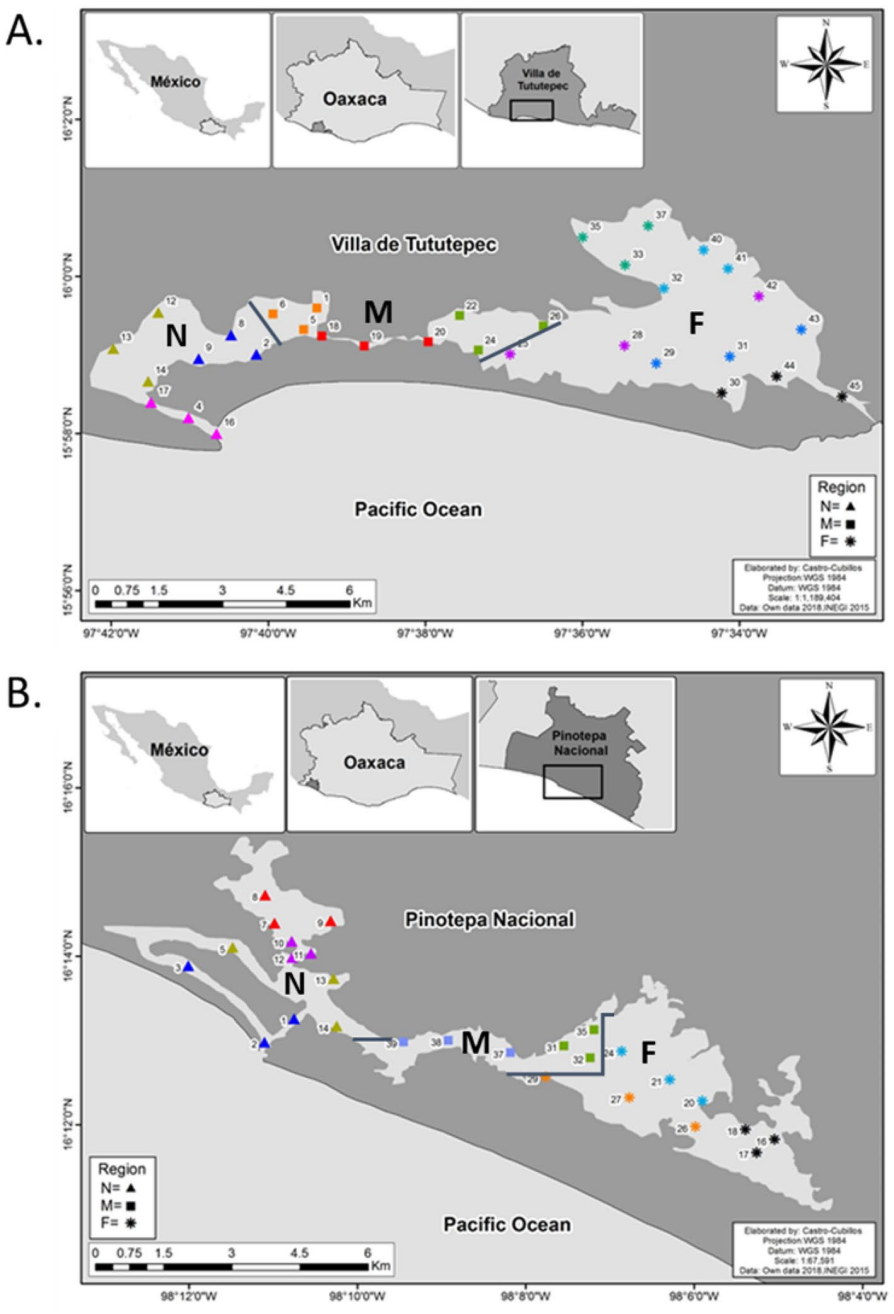
Study area and sampling locations in both lagoon systems. (**A**) CH, (**B**) C. Symbols indicate regions (filled triangle = near (N), filled square = middle (M), * = far (F)) and different colours indicate areas within regions. The maps in this figure were created with ArcGIS Desktop (Version 10.2), https://www.esri.com. They were generated with own data (sampling points) and data from INEGI (National Institute of Statistics and Geography), https://en.www.inegi.org.mx.

We chose two coastal lagoons of the Southern Mexican Pacific. The first one is Chacahua-Pastoria (CH) (15° 57′ 0.237′′–16° 03′ 05.96′′ N and 97° 31′ 57.15′′–97° 48′ 01.01′′ W) a RAMSAR site; and the second one is the Corralero-Alotengo system (C) (16° 11′ 15′′ and 16° 16′ 30′′ N and 98° 05′ 00′′ and 98° 12′ 30′′ W) along the Corralero fishing village which does not have a protected status even though both lagoon systems include mangrove ecosystems^42^ (Fig. 1). The climate of the region is tropical sub-humid with summer rainfall, from June to August. The salinity shows seasonality, presenting the highest values from February to June and the lowest from August to December^43^. There are marked differences between the mouth and the interior of the system in salinity and temperature; whereas the dissolved oxygen (DO) does not show clear spatial–temporal trends, and the pH shows extreme values in the dry season^37,43^.

Each lagoon was divided into different zones according to physicochemical (salinity, temperature, pH) and soil (sand, mud) characteristics, previously defined in other studies^34,43^. In total, there were 11 zones for CH and 9 zones for C, and three samples were taken from each zone. Finally, these areas were integrated into regions within the same lagoon systems; these regions were defined according to each lagoon’s distance to the mouth. The total distance of each lagoon body was taken and divided into three parts, resulting in the following regions: near, middle and far. For C, near (N): 0–4 km, middle (M): 4–8 km and far (F): 8–12 km; for CH, near (N): 0–7 km, middle (M): 7–14 km and far (F): 14–21 km.

Sediment samples were collected with a Petite Ponar grab twice in 2018. The first sampling period was towards the end of the dry season, March (20th to 22nd) and April (10th and 11th) and the second was at the end of the rainy season, September (17th to 20th). In each sampling location depth, salinity, temperature, pH and oxygen concentration were measured from the overlying water with a calibrated Hanna HI 9828 multi-parameter probe. All the samples were placed inside Ziploc bags and stored under ice until arrival at the laboratory. Each sample was homogenized with a blender that was cleaned with sodium hypochlorite between each use, finally, the sample was stored at − 20 °C until DNA extraction. The method of blending to homogenize sediment samples has been used previously (method outlined in Aylasgas et al.; Wangensteen et al.) and was conducted to homogenize as many organisms from a larger sample as possible, meaning DNA would be extracted from intact living organisms rather than DNA “traces”^22,44,45^.

### DNA extraction and sequencing

For DNA extractions, 10 g of each homogenized sediment sample were processed with the PowerMax DNA Isolation Kit (QIAGEN, Valencia, CA, USA) according to the manufacturer’s instructions, and DNA was resuspended in a final volume of 5 ml. The size of the DNA was confirmed with gel electrophoresis and each sample was quantified with a Nanodrop 2000.

The V4 region of the 18S rRNA gene was amplified with the forward primer E572F (5′-CYGCGGTAATTCCAGCTC-3′) and reverse primer E1009R (5′-AYGGTATCTRATCRTCTTYG-3′)^46^. To amplify a partial fragment of the mitochondrially encoded cytochrome c oxidase I (COI) gene, the forward primer mlCOIintF-XT (5′-GGWACWRGWTGRACWITITAYCCYCC-3′) and reverse primer jgHCO21 (5′-TAIACYTCIGGRTGICCRAARAAYCA-3′) were used^22^. Both primers contained the Illumina NextEra kit adapter and 12 random bases to increase diversity on the Illumina slide and the reverse primers contained the Illumina Nextera adapter.

The 18S-V4 amplicons had 25 µl final volume, with 0.625 U of GoTaq G2 Flexi DNA polymerase (Promega), 0.5 µl of 0.2 mM of each primer, 5 µl of Buffer 1×, 2 µl of MgCl_2_, (25 mM) 0.5 µl of dNTPs 10 mM and 1.5 µl (15 ng) of DNA template. For COI the final volume and concentrations were the same except for 25 mM of MgCl2 and 2 µl (15 ng) of DNA template. For 18S-V4, the PCR conditions consisted of a polymerase activation step of 5 min at 94 °C, 35 cycles of denaturation at 94 °C for 30 s, annealing at 50 °C for 45 s and elongation at 72 °C for 90 s, the final extension was for 72 °C for 7 min. For COI, the polymerase activation at 95 °C was for 7 min, followed by 46 cycles of denaturation at 94 °C for 30 s, annealing at 44 °C for 45 s and elongation at 72 °C for 90 s, the final extension was at 72 °C for 5 min.

PCR clean-up was performed with Agencourt AMPure beads at a concentration of 0.8× beads per sample. The DNA concentration of the PCR products was measured in a microplate reader FLUOstar Omega using the QuantiFluor dsDNA System (Promega, UK). 18S-V4 and COI amplicons were then pooled in equimolar concentrations and indexing was performed using an Illumina Nextera barcoding kit as per the manufacturer’s instructions. Indexed libraries were then pooled, quantified and diluted to 4 pM and ran on Illumina Miseq using the V3 2 × 300 bp Illumina sequencing kit (Illumina).

### Sequence processing and bioinformatics

The sequences were analysed with QIIME 1.9.1^47^ and the Usearch version 8.1^48^. The Usearch mergepairs command was used to assemble the raw paired ends sequences (forward and reverse). A quality filter was performed and the samples were separated out into 18S-V4 and COI datasets based on primer sequence using CUTADAPT^49^. Both primers, forward and reverse, were removed before further analysis. Sequences with lengths less than 370 bp for 18S-V4 and 300 bp for COI were removed, and sequences were quality filtered to remove those with maximum expected error > 0.5. Sequences were dereplicated, and any singleton sequences were removed, MOTUs were then clustered using the USEARCH UPARSE algorithm^50^ at 97% for both 18S-V4 and COI datasets. UCHIME^51^ was used to find and eliminate chimeras compared to the Silva (132 version) database^52^ for 18S-V4 and a custom database for the COI. The custom COI database was made from COI sequences downloaded from the NCBI (National Center for Biotechnology Information) (accessed 17/05/2018). Sequences were filtered to contain full length COI and partial fragments > 300 bp and no more than two consecutive N bases. The database was then dereplicated of identical sequences and clustered at 99% before both fasta files and taxonomy were manually formatted for input into QIIME. After removal of chimeras, MOTUs were classified against the reference databases using UCLUST with a minimum similarity of 0.6. COI sequences were also checked individually against the BOLD database^53^. Original reads were then mapped onto the MOTUs at 97% and a MOTU table was produced. The MOTUs sequences were checked against the full NCBI database at 100% for potential new records in the region.

### Statistical analysis

To analyze significant differences in the measured physicochemical parameters between regions, two-way ANOVAs and their post-hoc tests were performed (Tukey HDS), using the Stats v.3.5.2 package in R^54^. The metazoan and microeukaryotic MOTUs were separated out in each sample and the rarified absolute frequency matrix was used to perform diversity and statistical analyses; rarefaction curves were generated for each marker and richness estimators were evaluated in *Vegan* in R (Chao, Jacknife1, Jacknife2 and Bootstrap). For each lagoon system, the samples from each zone were pooled together and the abundances were adjusted by zone. For the metazoans, it was rarefied at 300 sequences and for the other eukaryotes at 3000 sequences (Supplementary Material Figs. S1, S5). For the alpha diversity, the richness of MOTUs was analysed in each zone. A one-way ANOVA and post-hoc test was done to verify if the differences found in the diversity measures (richness S) were significant, α = 0.05. To find the best correlation of richness within the 5 measured environmental parameters, the Pearson’s correlation index was also evaluated with the Stats V.3.5.2 package, both for metazoans and microeukaryotes.

To determine differences in the benthic community composition of the systems, a PERMANOVA^55^ was performed in Primer V7. Each data set was transformed to a presence/absence matrix to then apply a *Jaccard* similarity test to each one. With the resemblance result a 3 factor PERMANOVA design was applied, season (dry and rainy), lagoon system (CH and C) and region (distance to the lagoon mouth: near, medium and far). These analyses were run with 999 permutations and a p < 0.05. The region was nested in the lagoon system. A SIMPER analysis was done to obtain the groups that are responsible for the differences between the factors that showed significant differences in the PERMANOVA.

Subsequently, a canonical correspondence analysis (CCA)^56,57^ was carried out in PAST 3.25^58^ with the rarefied abundances of MOTUs for each zone within each lagoon system and the 5 environmental variables measured (salinity, temperature, DO, pH and depth) were measured to detect potential relationships between biological and environmental data.

## Results

### Lagoon conditions and environmental sampling

High variability in environmental parameters across season and within each lagoon were observed in both CH and C (Supplementary Material Table S1, Figs. S3, S4). In C, the depth varied between the three regions of the lagoon (ANOVA, df = 4, F = 5.18, p = 0.00149); the temperature varied between seasons (ANOVA, df = 1, F = 53.847, p = 2.23e−09) and between lagoon regions (ANOVA, df = 4, F = 5.455, p = 0.00106). Salinity showed a decrease in values in the three regions of the lagoon system from the dry to the rainy season (ANOVA, df = 1, F = 508.082, p = 2e−16), as well as differences between the lagoon regions in each season (ANOVA, df = 4, F = 6.747, p = 0.000216). pH varied significantly between seasons (ANOVA, df = 1, F = 72.86, p = 3.46e−11) and between regions (ANOVA, df = 4, F = 5.35, p = 0.00121), and DO varied between seasons (ANOVA, df = 1, F = 4,216, p = 0.0455) and between regions (ANOVA, df = 4, F = 13,500, p = 1.91e−07).

In CH, the depth was similar between seasons and regions of the lagoon, the temperature was different between regions of different seasons (ANOVA, df = 1, F = 77.88, p = 1.95e−12), and between regions of the same station (ANOVA, df = 4, F = 14.28, p = 3.03e−08). Salinity varied between regions within the same season (ANOVA, df = 4, F = 58.701, p = 2e−16). The pH varied between regions in different seasons (ANOVA, df = 1, F = 18.311, p = 6.85e−05) and between regions in the same season (ANOVA, df = 4, F = 5.964, p = 0.000415). DO varied between seasons (ANOVA, df = 1, F = 43.946, p = 1.07e−08) and between regions within the same season (ANOVA, df = 4, F = 6.147, p = 0.000326).

### Sequence metrics and comparisons between 18S V4 and COI

A total of 120 samples were analysed for each marker gene, 27 from C and 33 from CH for each season. For the 18S-V4 the initial number of raw sequences was 2,248,247. After read pair merging and the removal of short and low-quality reads control, the final number was 1,576,493 (70% remaining). For COI 1,758,686, raw reads were obtained and after the filtering, the final number was 319,417 (20% remaining) reads, with a high level of non-target amplification. After the MOTU clustering for 18S-V4 a total of 3410 MOTUs and 1182 for COI were detected. The COI marker dataset detected 16 phyla and the 18S-V4 marker 19 phyla. COI showed more metazoan MOTUs, despite V4 having more MOTUs overall. For this reason, COI was used to analyse the metazoan community in both lagoon systems. While COI and 18S V4 are standard barcodes for metazoans and eukaryotes, we found that several metazoan phyla were exclusively detected with 18S-V4 (Acanthocephala, Bryozoa, Ctenophora, Gnathostomulida and Tardigrada), while the Placozoa and Xenacoelomorpha phyla were only assigned in the samples analysed with COI; 13 phyla were assigned with the two markers (Annelida, Arthropoda, Chaetognatha, Chordata, Cnidaria, Echinodermata, Kinorhyncha, Mollusca, Nematoda, Nemertea, Platyhelminthes, Porifera and Rotifera).

### Taxonomic composition

**Figure 2:**
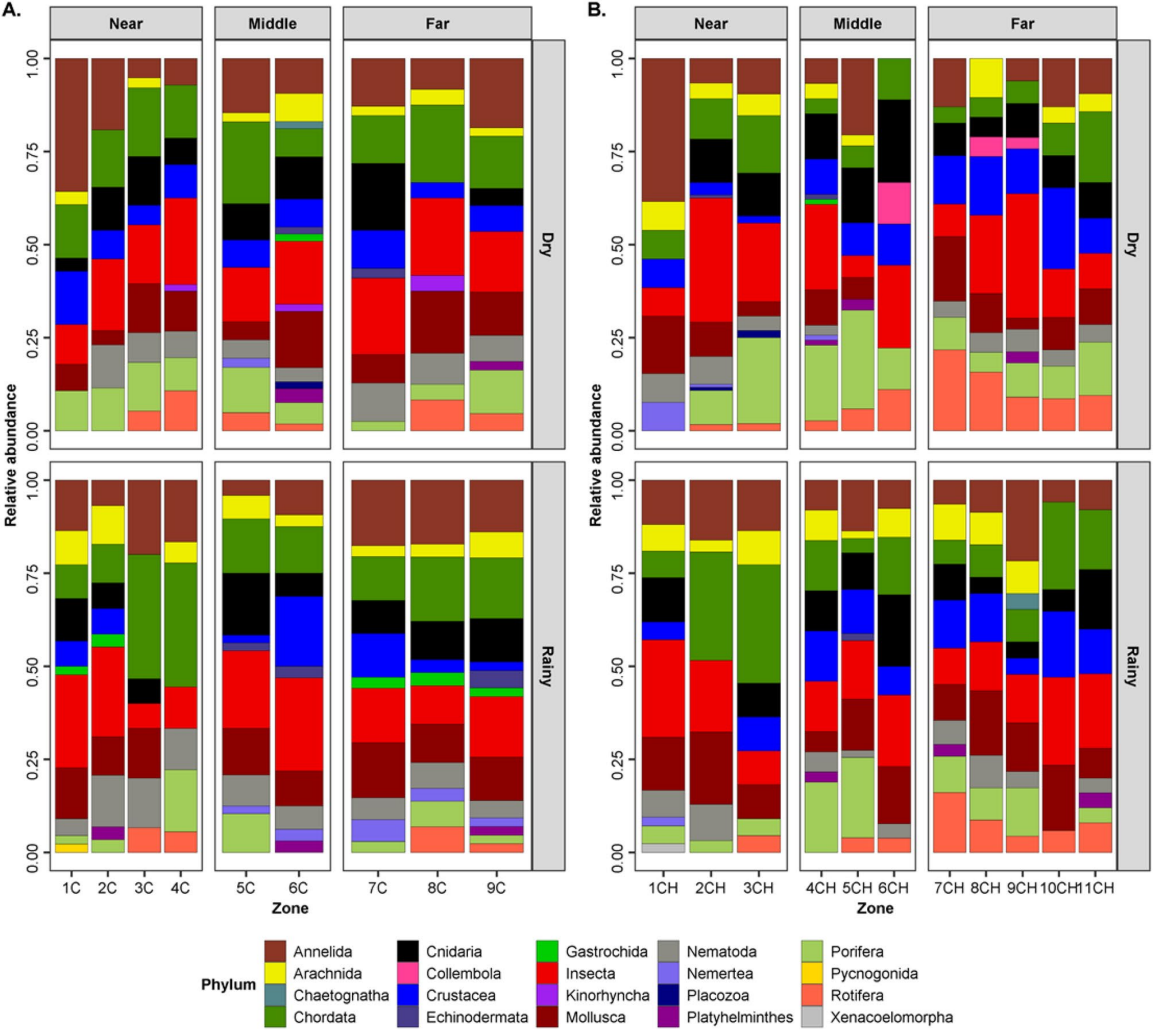
Relative abundance of MOTUs of Metazoan phyla in COI amplicon sequence libraries generated from sediments collected from Chacahua-Pastoria and Corralero-Alotengo lagoon systems throughout seasons, regions and lagoon zones. (**A**) C: Corralero-Alotengo Dry and Rainy seasons. Regions in the lagoon systems (distance to the mouth), N: Near (Zones: 1, 2, 3, 4), M: Middle (Zones: 5, 6) and F: Far (Zones: 7, 8, 9). (**B**) CH: Chacahua-Pastoria Dry and Rainy seasons. Regions in the lagoon systems (distance to the mouth), N: Near (Zones: 1, 2, 3), M: Middle (Zones: 4, 5, 6) and F: Far (Zones: 7, 8, 9, 10, 11).

**Figure 3:**
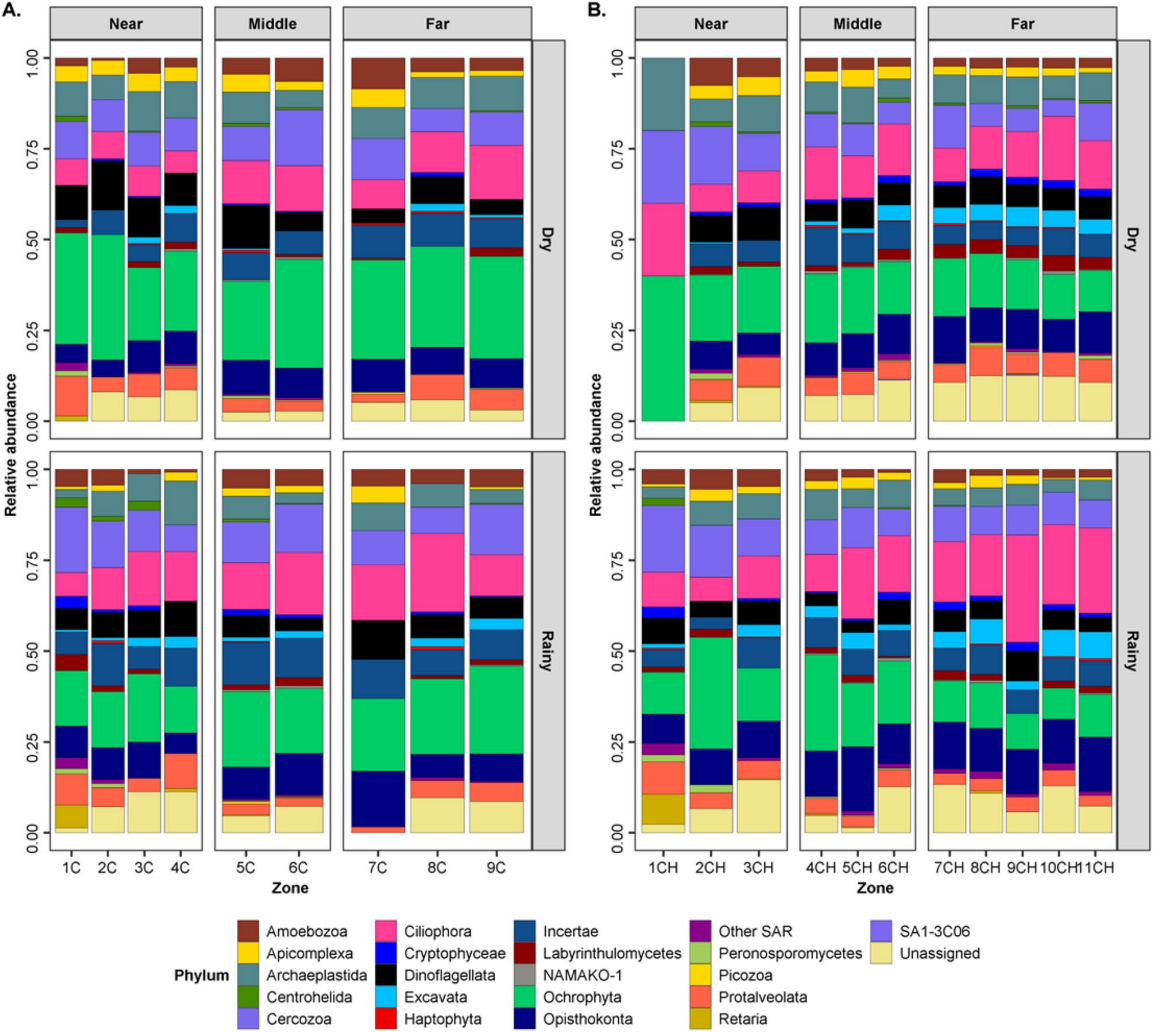
Relative abundance of MOTUs of microeukaryotic phyla in 18S-V4 amplicon sequence libraries generated from sediments collected from Chacahua-Pastoria and Corralero-Alotengo lagoon systems throughout seasons, regions and lagoon zones. (**A**) C: Corralero-Alotengo Dry and Rainy seasons. Regions in the lagoon systems (distance to the mouth), N: Near (Zones: 1, 2, 3, 4), M: Middle (Zones: 5, 6) and F: Far (Zones: 7, 8, 9). (**B**) CH: Chacahua-Pastoria Dry and Rainy seasons. Regions in the lagoon systems (distance to the mouth), N: Near (Zones: 1, 2, 3), M: Middle (Zones: 4, 5, 6) and F: Far (Zones: 7, 8, 9, 10, 11).

Arthropoda (31.1 ± 17.6% MOTUs) dominated in both lagoon systems and seasons. They were composed of Hexapoda (17.3% MOTUs ± 6.8), followed by Chordata (14.0% MOTUs ± 7.8), Annelida (12.4 ± 7.9% MOTUs), Mollusca (10.6 ± 4.8% MOTUs) primarily Bivalvia (5.3 ± 4.5% MOTUs) and Cnidaria (9.3 ± 5.2% MOTUs) (Fig. 2, Supplementary Material Table S2). Within the eukaryotic libraries, the most dominant taxa were the Ochrophyta (19.4 ± 7.3% MOTUs) primarily Diatomea (17.4 ± 7.4% MOTUs), Ciliophora (13.4 ± 5.18% MOTUs), Cercozoa (10.4 ± 3.5% MOTUs), Opisthokonta (9.6 ± 3.16% MOTUs), Archaeplastida (7.3 ± 3.05% MOTUs) and Dinoflagellata (6.6 ± 2.6% MOTUs) in each zone (Fig. 3).

Several MOTUs had close matches to taxa that had not been previously reported for these systems, namely some Crustacea and Dinoflagellata important in microalgal and potentially toxic blooms. With a sequence identity percentage of 100%, the barnacle *Amphibalanus eburneus* and the dinoflagellates *Alexandrium leei*, *Amphidinium klebsii* and *Gyrodinium jinhaense* were identified. Other closer matches were found, with sequence identity percentages of 98.5–100%, that match the crustaceans *Macrothrix* sp. and *Calanus propinquus,* and with the dinoflagellates *Lingulodinium polyedrum*, *Prorocentrum triestinum*, *Pellucidodinium psammophilum*, *Nusuttodinium amphidinioides*, *Alexandrium ostenfeldii*, *Alexandrium pohangense* and *Gonyaulax spinifera* (Supplementary Material Table S3). It is important to note the presence of bivalve species, *Mytella charruana* and *Mytella strigata* which are synonyms and species of commercial interest in the region^59,60^.

### Taxonomic richness in the lagoon systems

**Figure 4:**
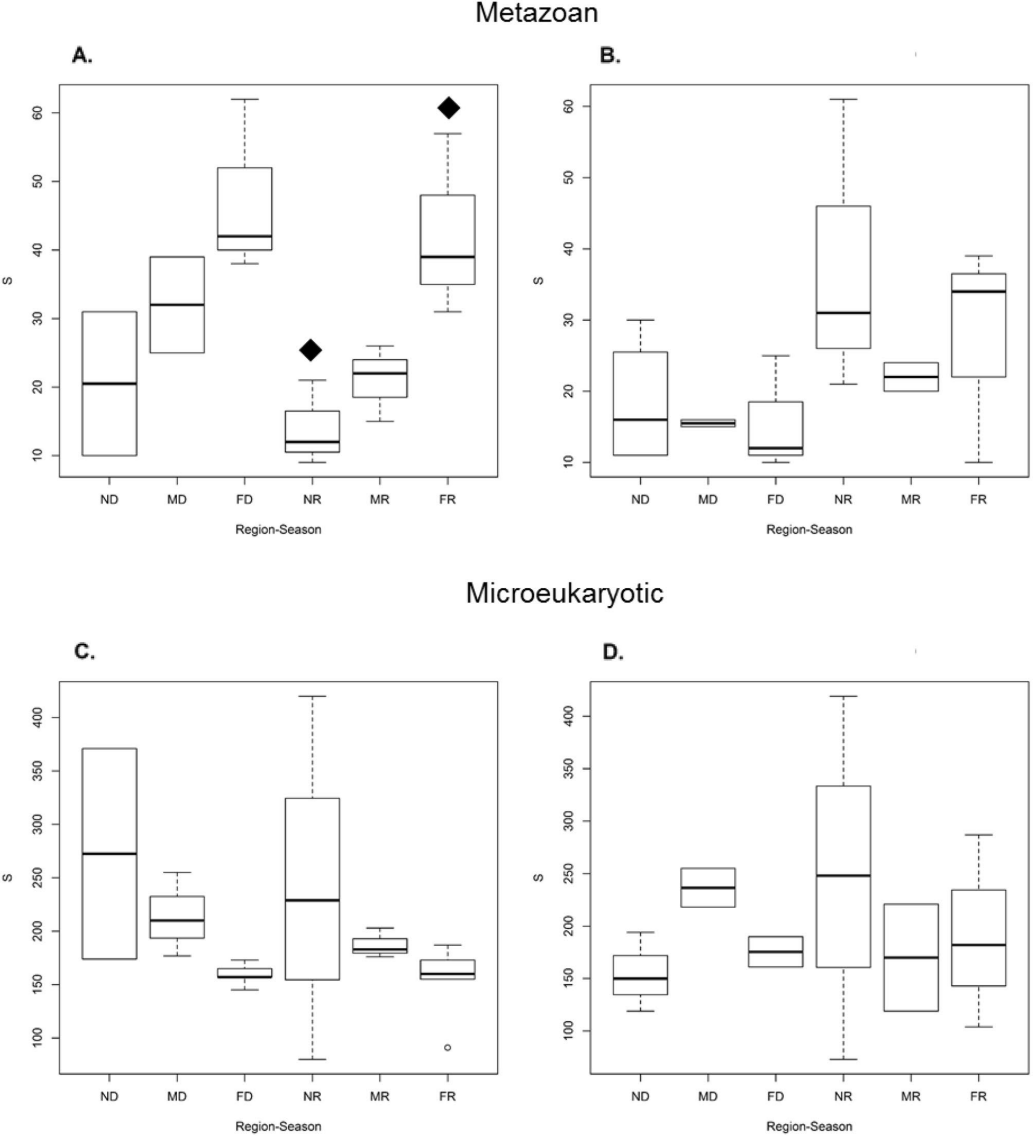
Boxplots for alpha diversity-richness (S) comparisons of metazoan and micro-eukaryotic communities between the lagoon systems: Metazoans (**A**) CH, (**B**) C; microeukaryotic (**C**) CH, (**D**) C. Filled diamond indicates significant differences between the pairs of regions analysed (ANOVA, F2,6 = 7.926, p = 0.0207), (Tukey HDS, p = 0.0205). On the x-axis (Season-Region) the first letter indicates the region (N, M and F) and the second letter indicates the season (D and R). On the Y axis, the richness of MOTUs is represented.

Of the 636 total metazoan MOTUs detected, 497 were from CH while 392 were detected in C. The richness of total MOTUs obtained in these samples was very close to that suggested by the richness estimators (Chao = 811.7, Jacknife1 = 835.3, Jacknife2 = 921.8 and Bootstrap = 729.5), between 69 and 87% (Supplementary Material Fig. S2). There was a trend of higher richness towards the region near the mouth during both seasons, while in CH it was in the region farthest from the mouth. Richness of MOTUs was higher in the dry season for C and for CH (Supplementary Material Figs. S3, S4). Significant richness differences were found between the CH regions during the rainy season (ANOVA, F2,6 = 7.926, p = 0.0207), specifically between the N-F regions (Tukey HDS, p = 0.0205), (Fig. 4). MOTUs richness showed moderate correlations with environmental factors such as salinity (Pearson, r =  − 0.69, p = 0.016) and pH (Pearson, r = 0.46, p = 0.0746) with highest correlation in CH (Supplementary Material Fig. S11); meanwhile, C showed a high correlation with temperature (Pearson, r = 0.45, p = 0.1087) and salinity (Pearson, r =  − 0.36, p = 0.0488) (Supplementary Material Fig. S12).

For microeukaryotes, MOTUs richness (S) was higher in both lagoon systems during the rainy season; being higher in CH (1818) than in C (1470) (Supplementary Material Figs. S7, S8). The richness of total MOTUs obtained in these samples was very close to that suggested by the richness estimators (Chao = 4225.3, Jacknife1 = 4151.4, Jacknife2 = 4759.7 and Bootstrap = 3519.67), between 71 and 85% (Supplementary Material Fig. S6). However, no significant differences were found globally among the regions of both lagoons in both seasons (Fig. 4). Meanwhile, the correlation of S with the environmental parameters was low. In CH, the parameter with the highest correlation was salinity (Pearson, r = 0.51, p = 0.1326) followed by temperature (Pearson, r =  − 0.41, p = 0.1907) and in C the highest correlations were with the OD (Pearson, r =  − 0.13, p = 0.0393) and pH (Pearson, r = 0.11, p = 0.8030) (Supplementary Material Figs. S13, S14).

### Composition and structure of the marine-coastal metazoan and eukaryotic community in both lagoon systems

**Figure 5:**
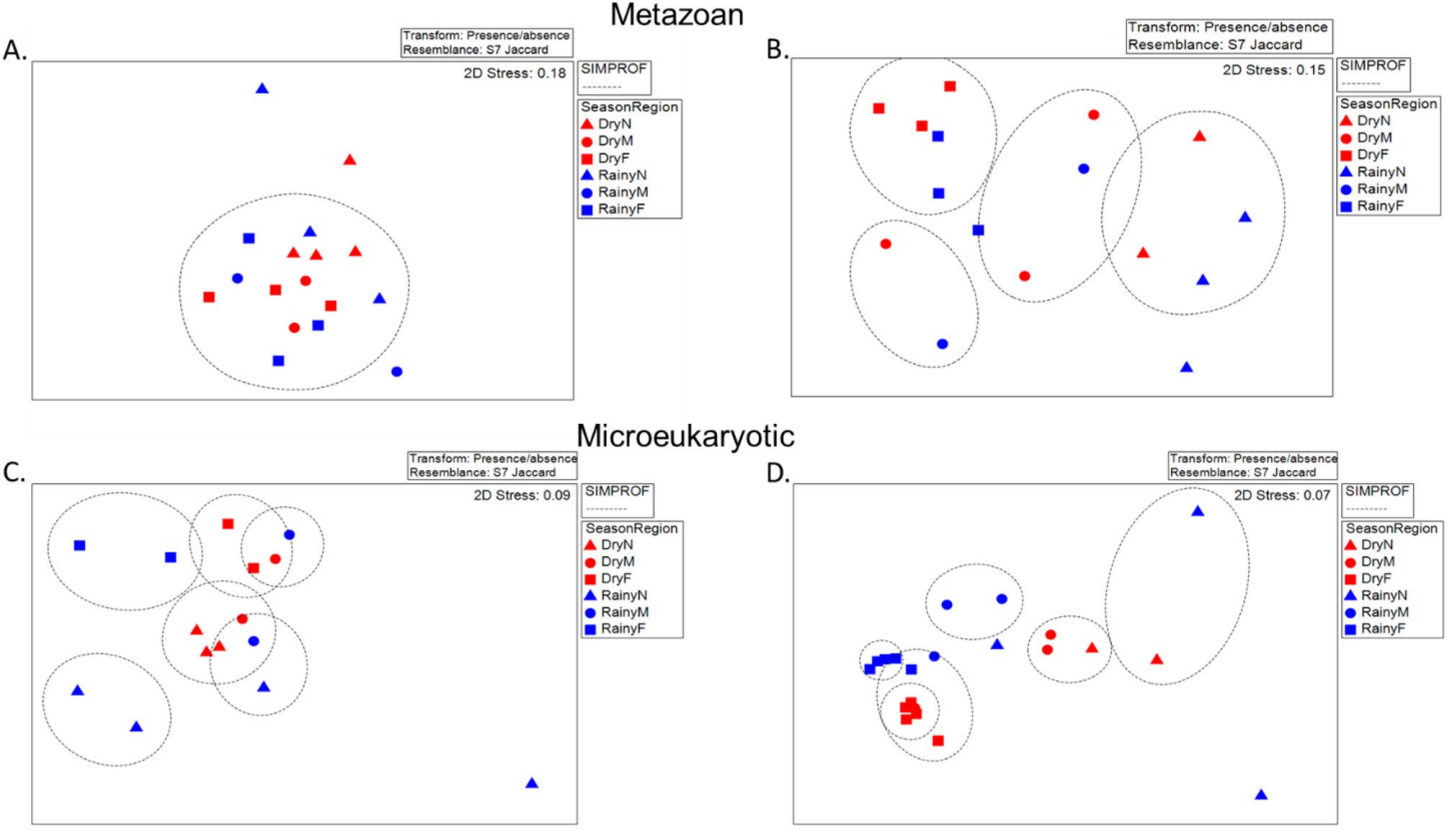
nMDS for metazoan and microeukaryotic communities identified in Corralero-Alotengo and Chacahua-Pastoria lagoon systems in both seasons. Metazoa: (**A**) C, (**B**) CH. Microeukaryotes: (**C**) C, (**D**) CH. All the representations have a stress value, for each one a presence/absence transformation and a Jaccard similarity test were applied. Groups were defined by the SIMPROF analysis.

Metazoan and microeukaryote community composition based on presence and absence of taxa showed no difference between both lagoon systems or in each lagoon system between seasons (PERMANOVA p > 0.05). After the pairwise comparisons, no significant differences were found between the C regions, but there were differences in CH for the metazoans; for CH, N-M (PERMANOVA df = 6, t = 1.2598, p = 0.047), N-F (PERMANOVA df = 7, t = 1.9294, p = 0.006) and M-F (PERMANOVA df = 7, t = 1.5704, p = 0.006). No differences in N, M, F metazoan communities were observed between seasons in either lagoon; nor between lagoons. For the microeukaryotes in C, the different regions were N-F (PERMANOVA df = 7, t = 1.239, p = 0.032); in CH the different regions were N-F (PERMANOVA df = 11, t = 1.8322 p = 0.002) and M-F (PERMANOVA df = 12, t = 1.4211 p = 0.005). In the dry season in CH, differences between M-F were observed (PERMANOVA df = 6, t = 1.299, p = 0.034), whereas in the rainy season between N-F (PERMANOVA df = 6, t = 1.427, p = 0.019) and M-F (PERMANOVA df = 6, t = 1.2311, p = 0.034). CH microeukaryotic community composition was similar in the far region F regardless of season, while microeukaryotic community composition was significantly different in M and N regions between seasons (Fig. 5). Comparing the same region across the two lagoons, the only significant differences were found in the rainy season with region F (PERMANOVA df = 6, t = 1.5022, p = 0.041).

Generally, the same metazoan phyla marked the differences between the seasons and the lagoon systems (molluscs, arthropods, nematodes, cnidarians and chordates). Arthropoda and Chordata were the main groups contributing to the differentiation between regions in both lagoon systems in both seasons, followed by Nematoda and Cnidaria in C and by Mollusca, Cnidaria and Annelida in CH, according to the SIMPER analysis of MOTUs relative abundance richness. Regarding microeukaryotes, the SIMPER analysis showed that Stramenopile diatoms made the largest contribution to the differentiation between regions, followed by Alveolata (Apicomplexa, Ciliophora, Dinoflagellata and Protalveolata) and Rhizaria (mainly, Cercozoa and to a lesser extent Rotaria) in C during both seasons, while in CH, the largest contribution was made by the organisms belonging to the SAR (Stramenopiles–Alveolata–Rhizaria) group, with Alveolata showing the highest values, followed by Stramenopiles and Rhizaria.

Metazoans showed a strong correlation with salinity (c = 0.95), temperature (c =  − 0.94) and pH (c =  − 0.83) in CH, while a gradient in the distribution of the community in the sediment influenced by pH (c =  − 0.86) and depth (c =  − 0.72) was identified in C in both seasons (Supplementary Material Fig. S9). Microeukaryotic community composition showed a greater correlation with temperature (c =  − 0.97), salinity (c = 0.96) and pH (c =  − 0.86) in CH (Supplementary Material Fig. S15). Meanwhile in C, a greater correlation of the distribution of organisms with pH (c =  − 0.92) was observed in the three regions in both seasons. The difference between seasons was marked by salinity (c =  − 0.97) and temperature (c = 0.96), thus, the regions in both lagoons had higher salinity values during the dry season than during the rainy season (Supplementary Material Fig. S15).

## Discussion

Metabarcoding has revolutionised the detection of benthic biodiversity^11,61–63^. Leveraging this method, the current study detected and documented the richness of metazoa and microeukaryotes in these lagoons, and in the wider region, for the first time. Taxonomic composition was similar to those of other metabarcoding studies in benthic environments, where the most abundant MOTUs correspond to Arthropoda, Mollusca, Annelida, Ochrophyta, Cercozoa and Ciliophora^11,17,27,64–66^. In this study, the recovery of eDNA from sediments revealed the presence of groups that are usually difficult to identify with the naked eye such as meiofauna, particularly Nematoda, and microeukaryotes^41,67^. Previous studies have shown that in aquatic systems, using metabarcoding to identify the benthic community, a greater number of MOTUs is obtained when using sediment samples compared to water samples^41^. A much higher richness of phyla was recovered using this method when compared to previous studies in these lagoon systems made with traditional taxonomy, which are focused in specific groups^34,37,38,68^. In comparison to many molecular studies, the approach used (homogenized sediment, combined with 10 g extraction) is likely to have captured most of the eukaryotic diversity within the analysed sediments; but without complete records of the metazoan and microeukaryotic communities in these lagoon systems, it’s unknown whether these samples are truly representative of the diversity of the entire lagoon system. However, the data collected for the current study considerably enhances the existing inventories^69^.

### Hidden biodiversity: new reports for coastal lagoons in the area

Species that have not been previously identified in the area were also detected; among the newly reported organisms, some were non-indigenous, invasive or harmful species, and others were bioindicators of the ecosystem’s health. The barnacle *Amphibalanus eburneus,* an invasive arthropod species native to the American Atlantic was found; in the Mexican Pacific it had already been reported in the Gulf of California, and further south^70,71^, the Corralero lagoon (this study) being the southernmost register so far. Within the copepods, the genus *Calanus* in CH was reported for the first time in the area; *Cletocamptus deitersi* had been previously reported in coastal lagoon systems and in estuaries in the coastal part of the northern Mexican Pacific Ocean in Sinaloa^72^; in the present study it was found only in the Corralero lagoon. The *Macrothrix* genus (Branchiopoda), is a cosmopolitan genus composed mostly of freshwater specimens and a few others that have ventured into marine environments, in plankton and benthos (associated with mud and debris); it has also been found in coastal lagoons and open waters often carried in vegetation^73–75^.

Although microeukaryotes have not been thoroughly studied in the area, there are records of some dinoflagellates of the genera *Alexandrium*, *Gymnodinium* and *Amphidinium* in the Mexican Pacific, which can cause human and animal intoxication via the consumption of shellfish containing these organisms^76,77^. These genera were identified for the first time in these lagoon systems in the current study, and could pose a risk to the Indigenous communities in the region for whom artisanal fisheries are a primary source of subsistence. Some of the dinoflagellates found in the sediments, mostly present in C and previously identified in the North of the Mexican Pacific^76,78^, represent a risk for aquaculture activities (shrimp farming and the fattening of fishes in captivity), marine megafauna and human health^79,80^, such as *Alexandrium leei, A. ostenfeldii, A. pohangense*, *Amphidinium klebsii, Prorocentrum triestinum*, *Lingulodinium polyedrum* and *Gonyaulax spinifera*^77,78,81^. Other non-toxic and grazer dinoflagellates were found too, *Gyrodinium jinhaense*, *Pellucidodinium psammophilum,* and *Nusuttodinium amphidinioides*^82,83^. The algal blooms in these lagoons may be associated with the resuspension of cysts from the sediment to the water column due to dredging, anthropogenic contamination, eutrophication and hypoxia that reaches these water bodies^84^. Furthermore, a total of 981 OTUs showed no 100% match to any reference sequences on the NCBI Genebank; this represents a great amount of biodiversity that is yet to be discovered and formally described.

### Taxonomic composition and structure of the marine-coastal metazoa and microeukaryote community and its relation with environmental lagoon conditions

The results obtained from the ANOVA show that the variations in the physicochemical parameters in the two lagoons over the two collection periods, show a typical characteristic of a coastal lagoon, where seasonal changes in the flow of rivers, changes in waves and tides, and meteorological variations, modify their salinity, temperature, dissolved oxygen, pH and depth^1,8^. Clear differences can be found between the areas near the mouth of the lagoon with marine influence, and far from the mouth of the lagoon with influence of rivers or freshwater sources. Being the middle zone, the transition zone between the other two areas, it is possible that here there is a mix of terrestrial, freshwater and marine MOTUs given the nature of the system. It is also plausible that we may have several MOTUs associated with individual taxa.

The distribution of benthic diversity in these lagoons has a strong relationship with environmental parameters, both lagoons show a transition from freshwater to seawater near the mouth of the system. This transition is not only observed in the physicochemical parameters, as it is more evident with salinity, but also with the biological community that inhabits the system. Salinity in these water bodies is the environmental characteristic that has the greatest effect on the biota^85^, however, it should be taken into account that these environmental variables do not necessarily act independently on organisms^86,87^. It is well known that salinity has an impact on the richness of crustaceans^9,88^, molluscs^86,89,90^ and cnidarians^91^; as well as in the microeukaryotic community, such as Ochrophyta (diatoms)^92^. There is a strong correlation between salinity and the protist community in the coastal lagoons, generating a gradient in diversity^93–95^. Distinct differences in richness of taxa between the lagoons was observed, with CH being richer in metazoan and microeukaryotes MOTUs than C, considering the two seasons. Our results show that although the two lagoon systems are similar in terms of the composition of metazoans and microeukaryotes, they are different in relative abundance in each lagoon, resulting in regional partitioning of the diversity for CH. This regional partitioning is clearer in the dry season, and salinity seems to be the driving factor in community composition. The dominant taxa driving this dissimilarity between regions were Arthropoda (148 MOTUs), Cnidaria (33 MOTUs), Mollusca (32 MOTUs) and Annelida (30 MOTUs). In other Mexican coastal lagoons, the diversity of marine metazoans (vertebrates and invertebrates) is usually slightly higher in groups such as Mollusca, Crustacea and Polychaeta^96^. However, the lower diversity presented in C can be explained by the varied environmental conditions due to shallow depth and restricted communication with the sea, as has been observed in Mediterranean lagoons^97^. Clearly, for C the diversity in the region near the mouth of the lagoon was greater in the two seasons for the metazoans and microeukaryotes; in CH, a clear predominance of marine species was observed in microeukaryotes, whereas in metazoans brackish species predominate. This result shows that the values of diversity indices tend to decrease as the degree of confinement within the lagoons increases^97^.

Even though the composition of taxa in the two systems is very similar, differences are found in the total number of MOTUs of metazoans and microeukaryotes in the two lagoon systems which could have been down to the differences in the sampling effort for both lagoons. The protected system (CH) is more diverse, with clear differences in the composition between regions due to their environmental differences. Clearly, the middle region of CH represents the transition, both physiochemically and biologically, between the marine and brackish environments. The opposite occurs in C, where the environmental characteristics are more homogeneous between regions at each time throughout the lagoon system. This may be due to the more restricted communication with the sea. Therefore, when the mouth of the lagoon is closed, it has a bigger influence of freshwater, which drastically changes the environmental configuration of the system and consequently the distribution of the biological community. Another factor that can directly affect diversity in C is the dredging carried out with high periodicity and the deposition of sediments in different parts of the system. Dredging moves organisms from one region to another, causing the loss of diversity that a heterogeneous environment usually provides. This effect is more evident in the metazoan community, which includes groups of commercial and alimentary importance, evidencing the benefits of the partial state of conservation of the CH system and the importance of Ramsar sites.

Although the comparison between previous studies and the present was one of our main research questions in order to give perspective to the efficiency of the metabarcoding technique, this has proven to be very difficult due to a couple of reasons. Previous biodiversity studies in Chacahua have focused primarily on certain species of invertebrates (polychaetes and molluscs), so the comparison would be limited only to these taxa^98,99^. Secondly, when doing this comparison of species, we found only a few coincidences of conspicuous species such as *Mytella strigata* (mollusc of commercial importance). These could be mainly because although through the years there has been several efforts to characterise the biodiversity of these groups in the area, these efforts have not been accompanied by the genetic characterisation of the specimens, therefore a species reference library is not available, for this reason some of the BLAST hits would not be able to find the exact species and will give the closest register they have. Another reason is that some of the species have changed names, for example *Tryonia robusta* is now *Ipnobius robustus* and *Neritina granosa* is now *Neritona granosa*, these changes in taxonomic situation are difficult to identify if you are not a specialist in the group. Regarding polychaetes, several genera have already been registered in the Southern Mexican Pacific such as Branchiomma, Neanthes, and Polydora, but they include numerous species that most likely have not been sequenced. There are other groups of meiofauna annelids that have not been studied in the Southern Mexican Pacific at all (Doliodrilus, Thalassodrilides, Pontoscolex, Protodrilus), our results becoming their first registers.

In conclusion, eDNA metabarcoding of sediments has proven an important contribution to the documentation of relevant species and groups in tropical coastal lagoons expanding the information contained in databases for a very biodiverse area. It also provides insight into the space–time landscape of this type of ecosystem and reveals the presence of some groups of metazoans and microeukaryotes that had not been previously registered in Mexico’s coastal lagoons, such as oligochaetes, rotifers, nematodes, gills, turbellarians, cestodes and bryozoans. It was also possible to observe the regional partitioning of diversity in both lagoons depending on the distance from the mouth (entry of marine water and changes in salinity). Although spatial differences are observed in the two lagoon systems at two different times, there is a clear regional partitioning of the diversity (in terms of dominant groups). In order to establish a clear seasonal pattern, it is recommended that future studies extend the time and/or the periodicity of the samplings. Despite this limitation of the current study, a temporal component can be seen in both lagoon systems.

The species registered in this study and the reproducibility of this technique in future samplings will allow increasing knowledge and monitoring of the diversity dynamics of these ecosystems, since some are of fishing, commercial and bioprospecting importance. It is also crucial to monitor the presence of previously undetected groups that could be related to anthropogenic impact such as parasites, invasive species and toxic algal blooms. For all these reasons, metabarcoding turns out to be a fairly efficient means to monitor biodiversity, providing fast information on the distribution and abundance of species in a changing world where the rate of loss and extinction may surpass our understanding of it, especially in communities where small size organisms are found^66,100,101^. However, eDNA metabarcoding should go hand in hand with traditional taxonomy to improve the development of more precise barcoding reference libraries that will allow for a more efficient biomonitoring and species inventory especially in areas of great diversity with the potential to find new species^102^.

## Supplementary Information

Supplementary Information 1.

Supplementary Information 2.

Supplementary Information 3.

Supplementary Information 4.

Supplementary Information 5.

Supplementary Information 6.

Supplementary Information 7.

Supplementary Information 8.

## Data Availability

Raw sequence data was uploaded to the European Nucleotide Archive under the project accession: PRJEB52950. Environmental data and OTU tables are uploaded as Supplementary Information.
