# Supplementary material for: Monitoring of benthic eukaryotic communities in two tropical coastal lagoons through eDNA metabarcoding: a spatial and temporal approximation
Source: Sci Rep. 2022 Jun 16;12:10089. doi: 10.1038/s41598-022-13653-9 (PMC9203746; doi:10.1038/s41598-022-13653-9)

**Supplementary material for:**

Monitoring of benthic eukaryotic communities in two tropical coastal lagoons through eDNA metabarcoding: a spatial and temporal approximation

Margoth L. Castro-Cubillos ^a^, Joe D. Taylor ^b, c^, Alicia Mastretta-Yanes ^d, e^, Francisco Benítez-Villalobos ^f^, Valentina Islas-Villanueva ^e, g, *^

a. Programa de Doctorado en Ecología Marina, División de Estudios de Posgrado, Universidad del Mar Campus Puerto Ángel, Cd. Universitaria s/n, Oaxaca, 70902, México. lilianacastrocubillos@gmail.com ORCID:0000-0002-6233-1572

b. School of Environment & Life Sciences, University of Salford, Peel Building, Salford, M5 4WT, UK*.*drjoetay@gmail.com ORCID: 0000-0003-0095-0869

c. UK Centre for Ecology & Hydrology, Maclean Building, Benson Lane, Crowmarsh Gifford, Wallingford, OX10 8BB, UK

d. Comisión Nacional para el Conocimiento y Uso de la Biodiversidad (CONABIO), México. amastretta@conabio.gob.mx ORCID: 0000-0003-2951-6353

e. Consejo Nacional de Ciencia y Tecnología (CONACYT), México.

f. Universidad del Mar, Instituto de recursos, campus Puerto Ángel, Oaxaca, México. fbv@angel.umar.mx ORCID: 0000-0002-0951-7757

g. Universidad del Mar, Instituto de Genética, campus Puerto Ángel, Oaxaca, México. valentina@angel.umar.mx ORCID: 0000-0001-8829-3052

**Corresponding author**

Correpondence to Valentina Islas-Villanueva valentina@angel.umar.mx

**Table S1.** Environmental data summary by lagoon, season and region. Mean, Standard deviation (SD), minimum (min) and maximum (max) values per season for each region (N:Near, M: Middle, F: Far). DO: dissolved oxygen.

| Lagoon-season | Region | Depth (m) | Temperature °C | Salinity PSU | pH | DO (mg/l) |
| --- | --- | --- | --- | --- | --- | --- |
| Corralero Dry | N | 2.86±1.46 (0.42) | 31.56±1.57 (0.45) | 40.48±2.23 (0.64) | 8.29±0.27 (0.08) | 5.98±0.56 (0.16) |
|  |  | 1.2-5.3 | 28.56-32.85 | 36.35-42.88 | 8.03-8.91 | 4.9-7.06 |
|  | M | 2.17±1.18 (0.48) | 31.47±0.21 (0.09) | 42.42±1.44 (0.59) | 8.36±0.13 (0.05) | 5.12±0.93 (0.38) |
|  |  | 0.7-3.5 | 31.16-31.65 | 40.84-43.99 | 8.19-8.52 | 3.55-5.98 |
|  | F | 1.16±0.78 (0.26) | 30.61±0.83 (0.28) | 44.94±1.72 (0.57) | 8.23±0.08 (0.03) | 3.94±0.7 (0.16) |
|  |  | 0.3-3.0 | 29.15-31.64 | 42.3-47.65 | 8.13-8.33 | 2.91-4.95 |
| Corralero Rainy | N | 2.88±1.44 (0.41) | 33.74±0.64 (0.19) | 29.41±3.5 (1.01) | 8.03±0.15 (0.04) | 5.46±0.68 (0.20) |
|  |  | 0.4-5.6 | 32.48-34.65 | 23.27-33.65 | 7.64-8.27 | 3.4-6.03 |
|  | M | 2.44±1.24 (0.56) | 32.92±0.59 (0.26) | 29.2±1.78 (0.79) | 7.76±0.26 (0.12) | 4.41±0.95 (0.42) |
|  |  | 0.95-4 | 32.2-33.77 | 27.54-31.2 | 7.33-8.03 | 2.7-5.34 |
|  | F | 1.26±0.35 (0.12) | 32.17±0.21 (0.07) | 26.92±0.85 (0.28) | 7.7±0.11 (0.04) | 3.71±1.22 (0.41) |
|  |  | 0.75-1.75 | 31.81-32.43 | 25.6-28.05 | 7.54-7.85 | 1.95-5.43 |
| Chacahua Dry | N | 3.19±3.45 (1.15) | 25.97±3.29 (1.10) | 36.54±0.29 (0.10) | 8.21±0.15 (0.05) | 5.39±0.45 (0.15) |
|  |  | 1.1-12 | 21.67-29.66 | 36.16-37.11 | 7.99-8.48 | 4.8-6.05 |
|  | M | 1.66±0.82 (0.27) | 29.16±1 (0.33) | 27.23±9.27 (3.09) | 7.99±0.41 (0.14) | 5.23±2.51 (0.84) |
|  |  | 0.45-3.1 | 27.5-30.39 | 17.16-36.36 | 7.35-8.48 | 0.94-8.53 |
|  | F | 2.29±0.78 (0.20) | 30.31±0.57 (0.15) | 16.11±0.57 (0.15) | 8.57±0.4 (0.10) | 7.91±1.91 (0.49) |
|  |  | 0.7-3.85 | 29.34-31.47 | 15.23-17.53 | 8.17-9.85 | 6.06-14.38 |
| Chacahua Rainy | N | 3.25±3.66 (1.22) | 31.97±0.57 (0.19) | 35.91±0.84 (0.28) | 7.92±0.38 (0.13) | 4.57±0.64 (0.21) |
|  |  | 1.35-12.8 | 31.08-32.79 | 34.34-36.65 | 7.28-8.55 | 3.3-5.26 |
|  | M | 2.24±0.9 (0.30) | 32.06±0.76 (0.25) | 26.76±6.65 (2.22) | 7.77±0.41 (0.14) | 4.06±2.31 (0.77) |
|  |  | 1.2-3.98 | 30.84-32.98 | 19.59-34.1 | 7.26-8.27 | 0.74-7.67 |
|  | F | 2.59±0.77 (0.20) | 31.58±0.77 (0.20) | 18.54±0.67 (0.17) | 8.1±0.18 (0.05) | 2.9±1.46 (0.38) |
|  |  | 1.4.1 | 30.35-32.63 | 17.11-20.01 | 7.61-8.31 | 0-5.02 |

**Table S2.** Metazoan MOTUs per phylum in each lagoon

| Phylum | Total MOTUs | Chacahua MOTUs | Corralero MOTUs |
| --- | --- | --- | --- |
| Arthropoda | 148 | 110 | 81 |
| Cnidaria | 33 | 26 | 22 |
| Mollusca | 32 | 26 | 21 |
| Annelida | 30 | 23 | 23 |
| Porifera | 25 | 23 | 15 |
| Chordata | 24 | 20 | 18 |
| Nematoda | 15 | 10 | 10 |
| Rotifera | 9 | 7 | 8 |
| Echinodermata | 3 | 2 | 2 |
| Nemertea | 3 | 2 | 2 |
| Platyhelminthes | 3 | 1 | 3 |
| Chaetognatha | 2 | 1 | 1 |
| Placozoa | 2 | 1 | 1 |
| Gastrochida | 1 | 1 | 1 |
| Kinorhyncha | 1 | 0 | 1 |
| Xenacoelomorpha | 1 | 1 | 0 |

**Fig. S1** Rarefaction curves for the metazoan community in samples of both lagoon systems per number of sequences.


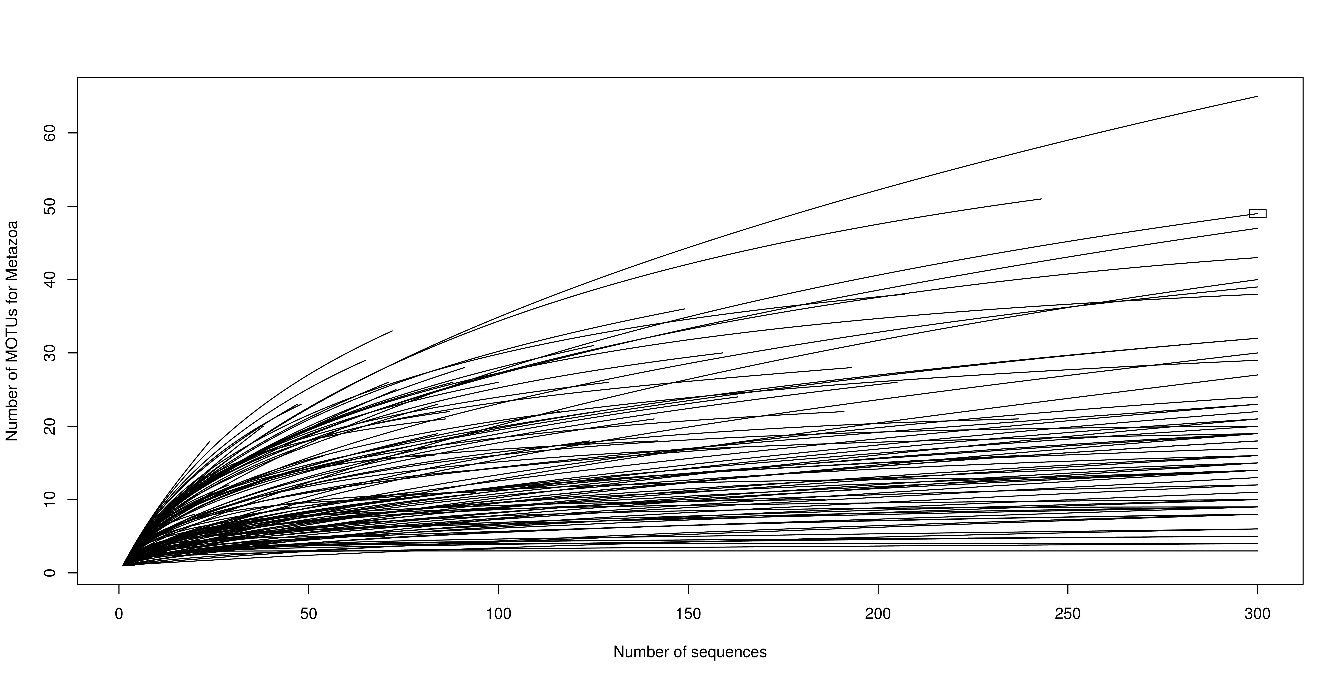


**Fig. S2** Rarefaction curves for the metazoan community per sample in both lagoon systems. Number of MOTUs per sample.


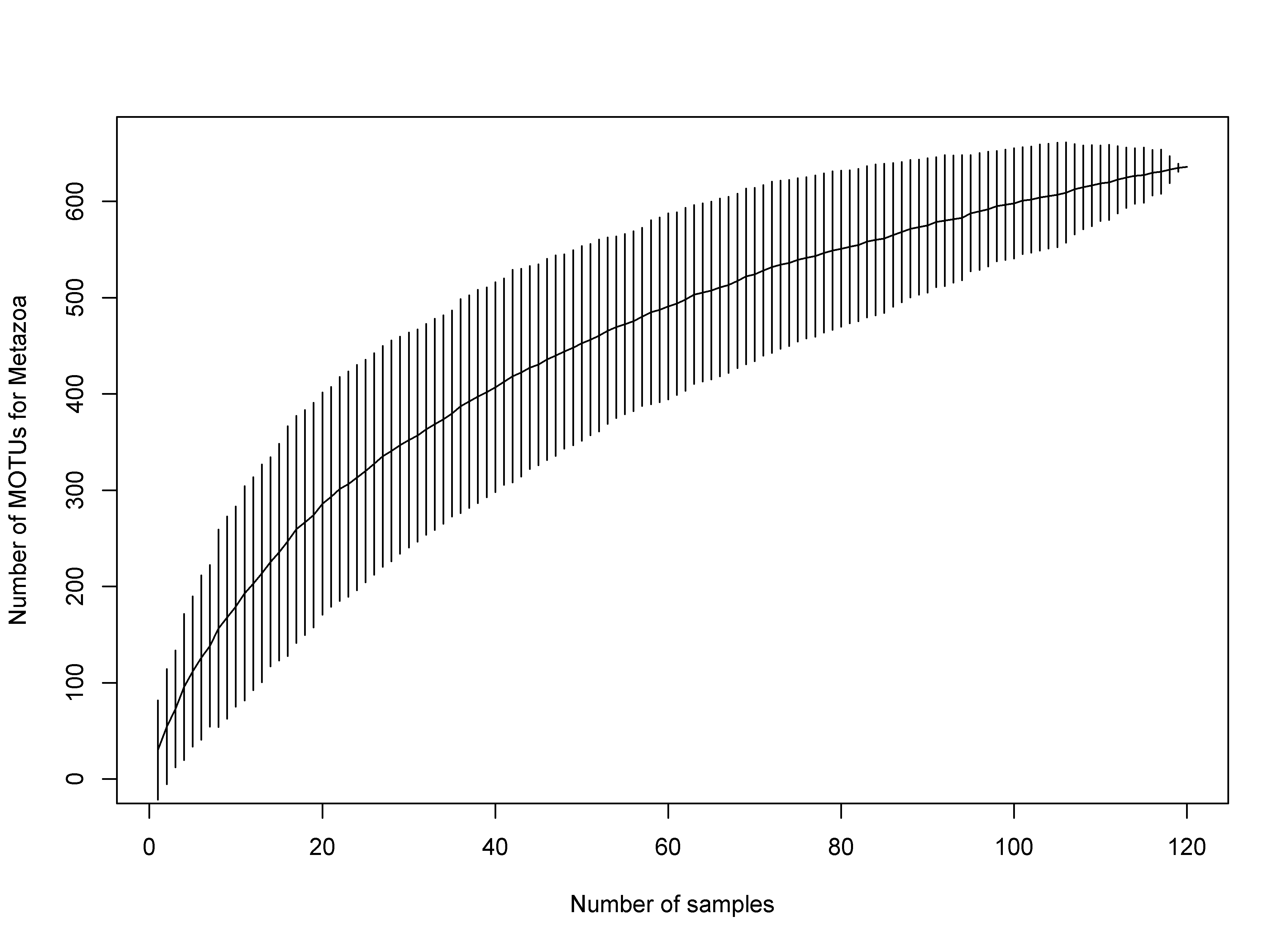


**Fig. S3** Rarefaction curves for the metazoan community in Corralero-Alotengo. Number of MOTUs per sample in each season: A. Dry season, B. Rainy season.


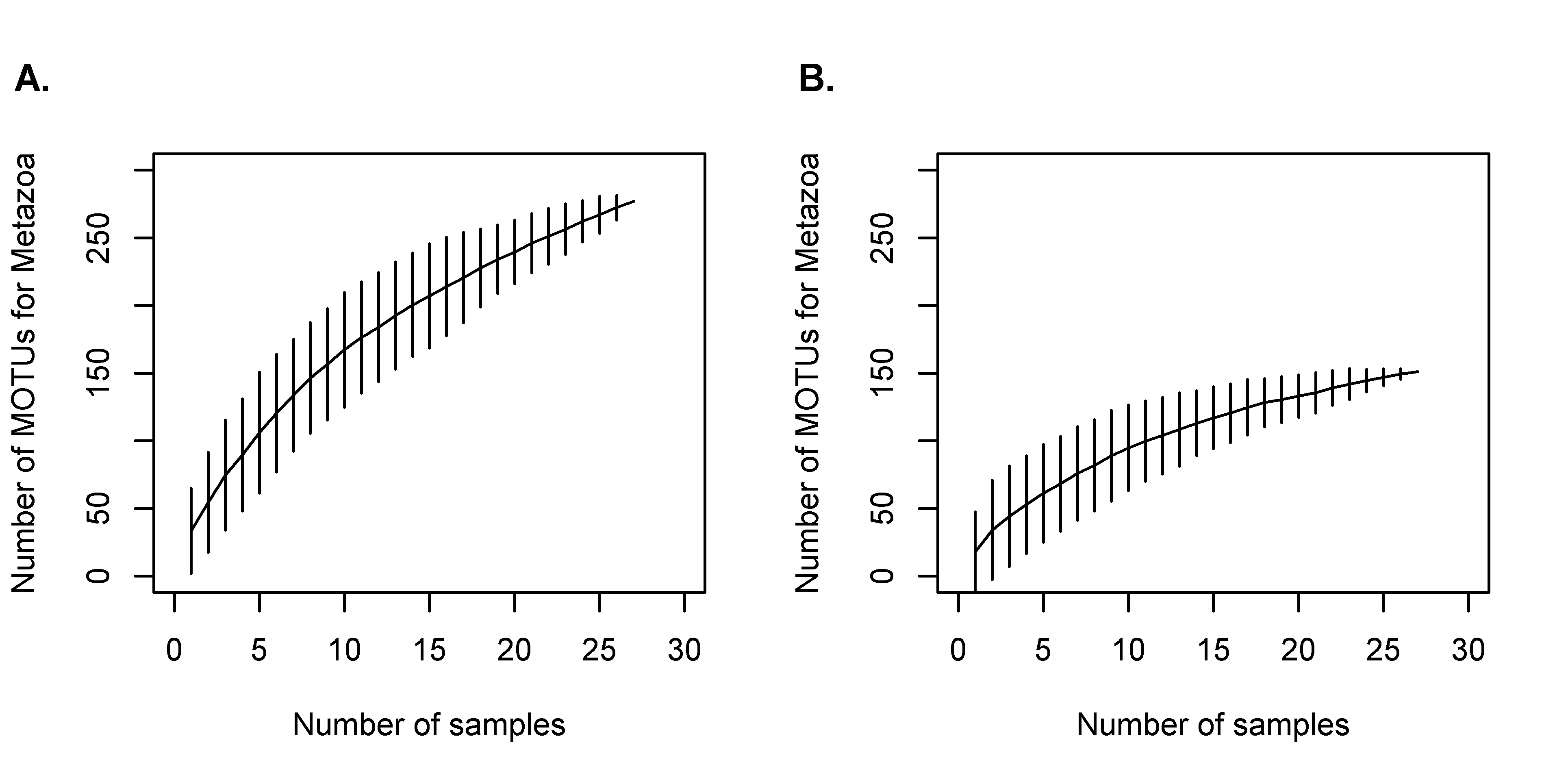


**Fig. S4** Rarefaction curves for the metazoan community in Chacahua-Pastoria. Number of MOTUs per sample in each season: A. Dry season, B. Rainy season.


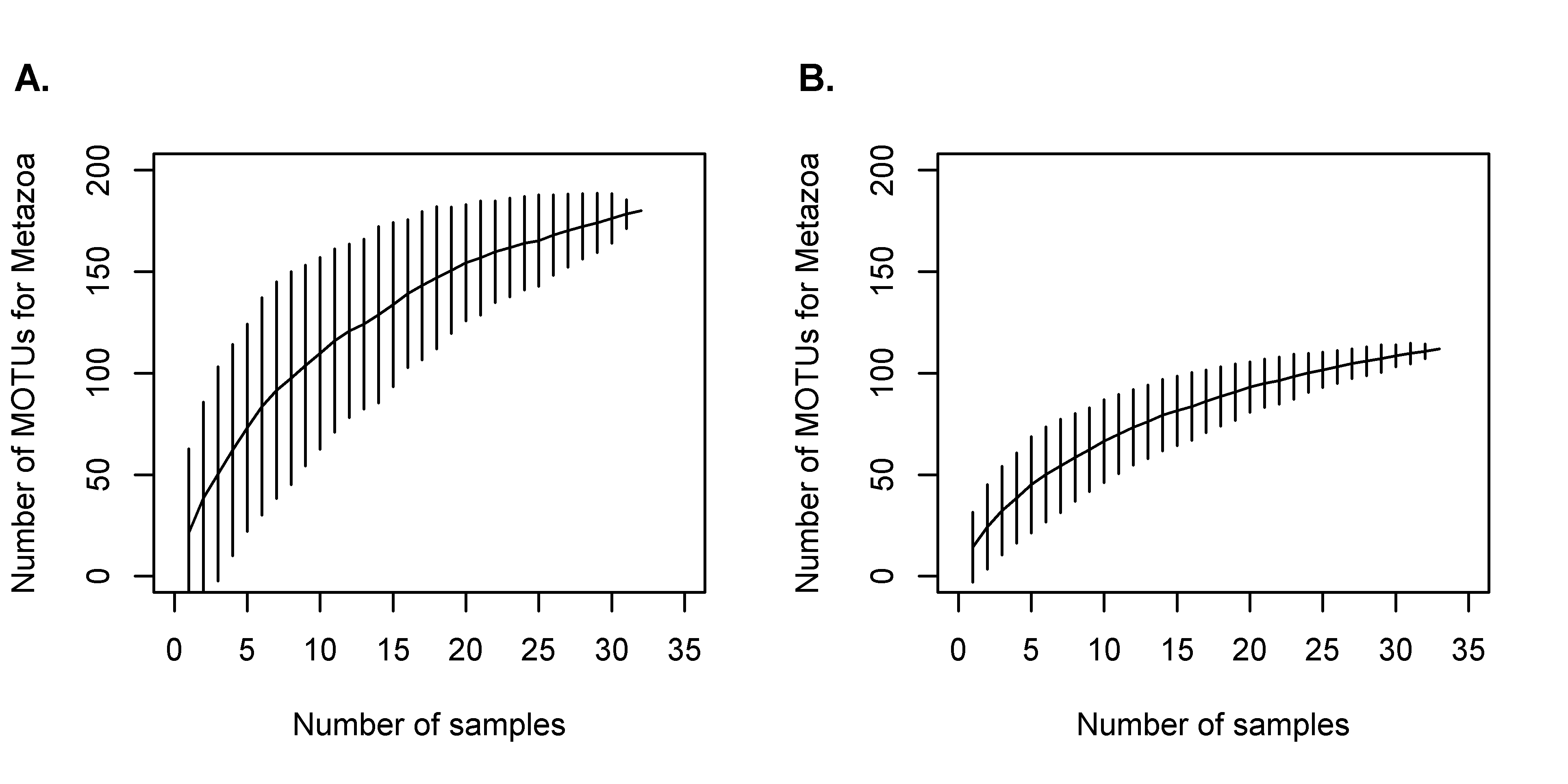


**Fig. S5** Rarefaction curves for the micro eukaryotic community in samples of both lagoon systems per number of sequences.
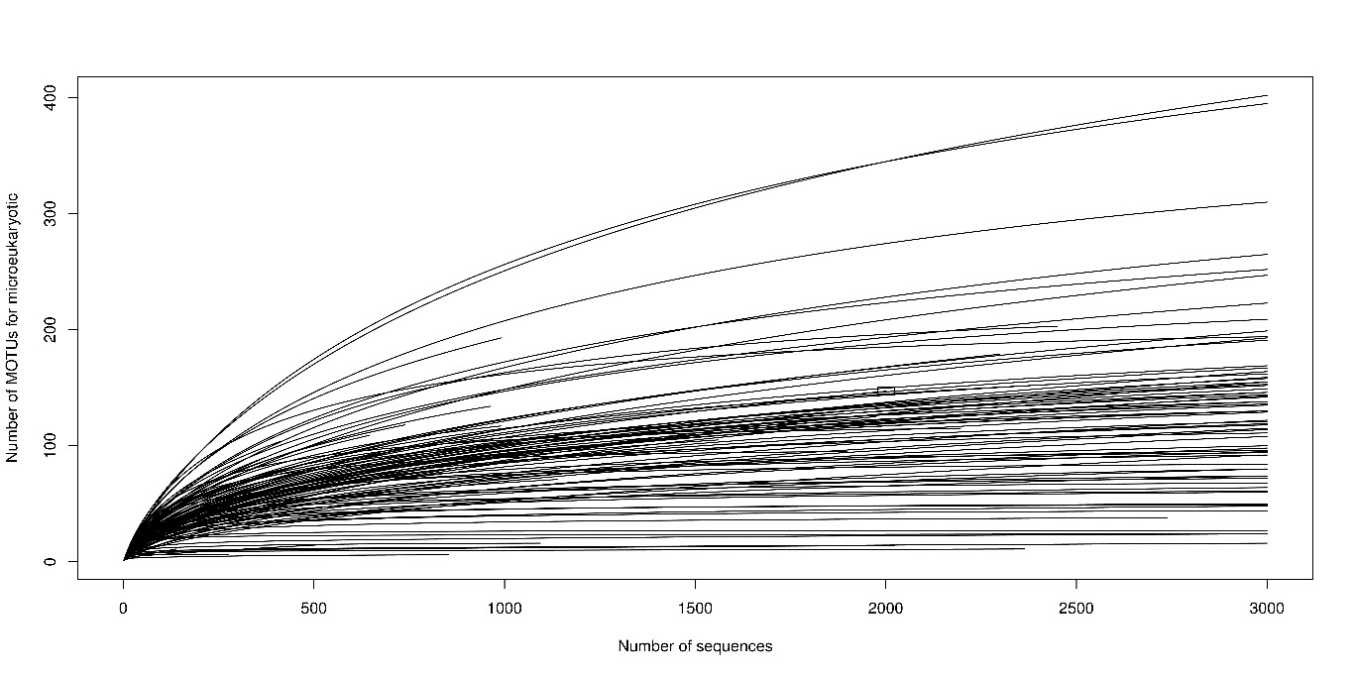


**Fig. S6** Rarefaction curves for the micro eukaryotic community per sample in both lagoon systems. Number of MOTUs per sample.


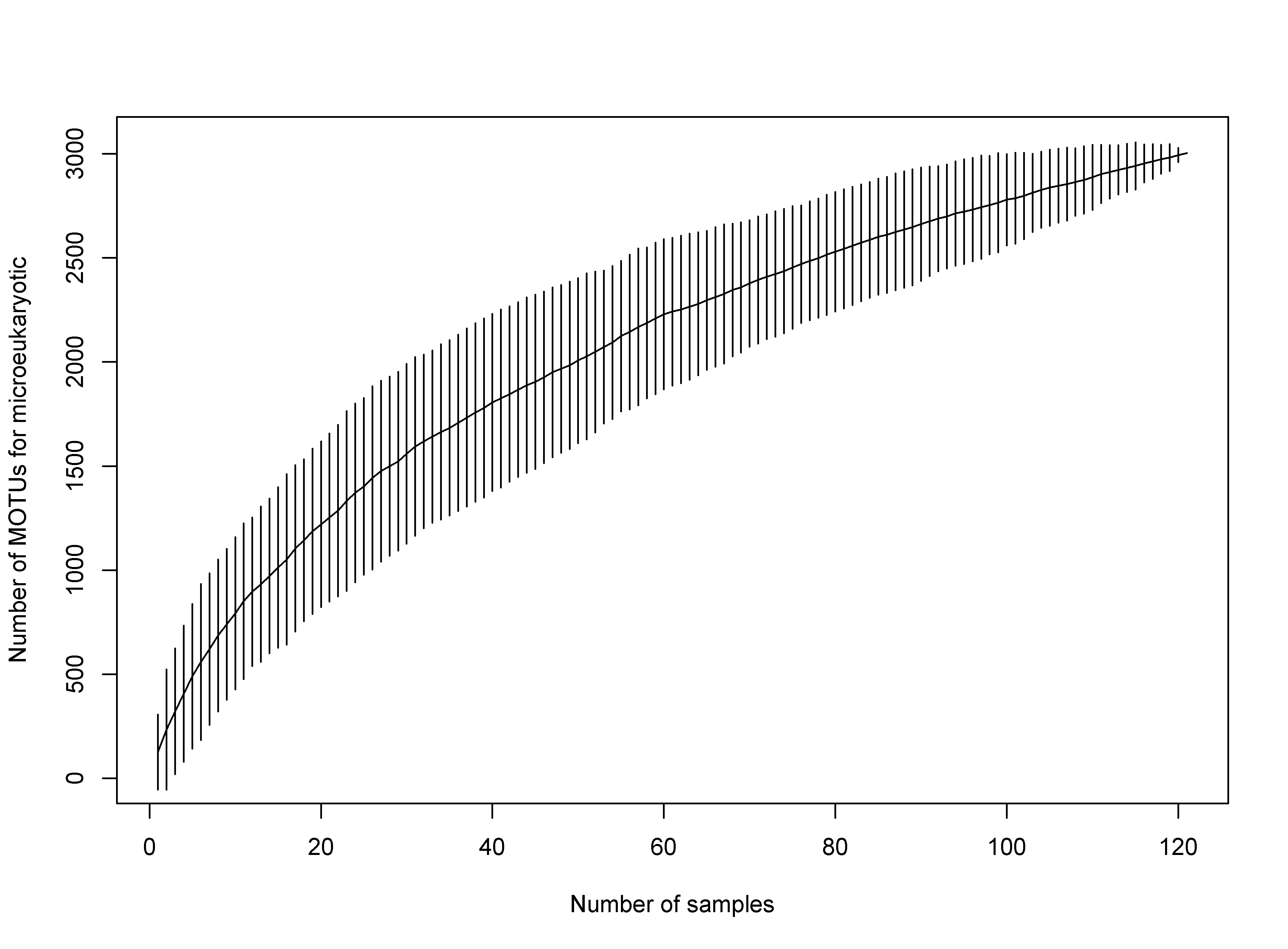


**Fig. S7** Rarefaction curves for the microeukaryotic community in Corralero-Alotengo. Number of MOTUs per sample in each season: A. Dry season, B. Rainy season.


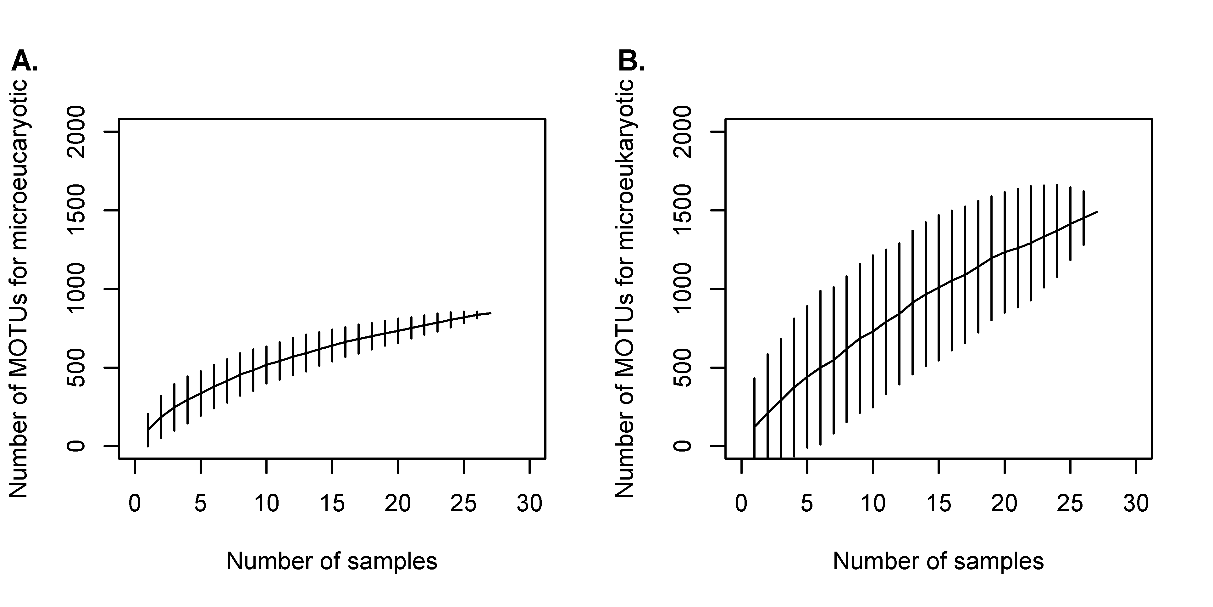


**Fig. S8** Rarefaction curves for the metazoan community in Chacahua-Pastoria. Number of MOTUs per sample in each season: A. Dry season, B. Rainy season.


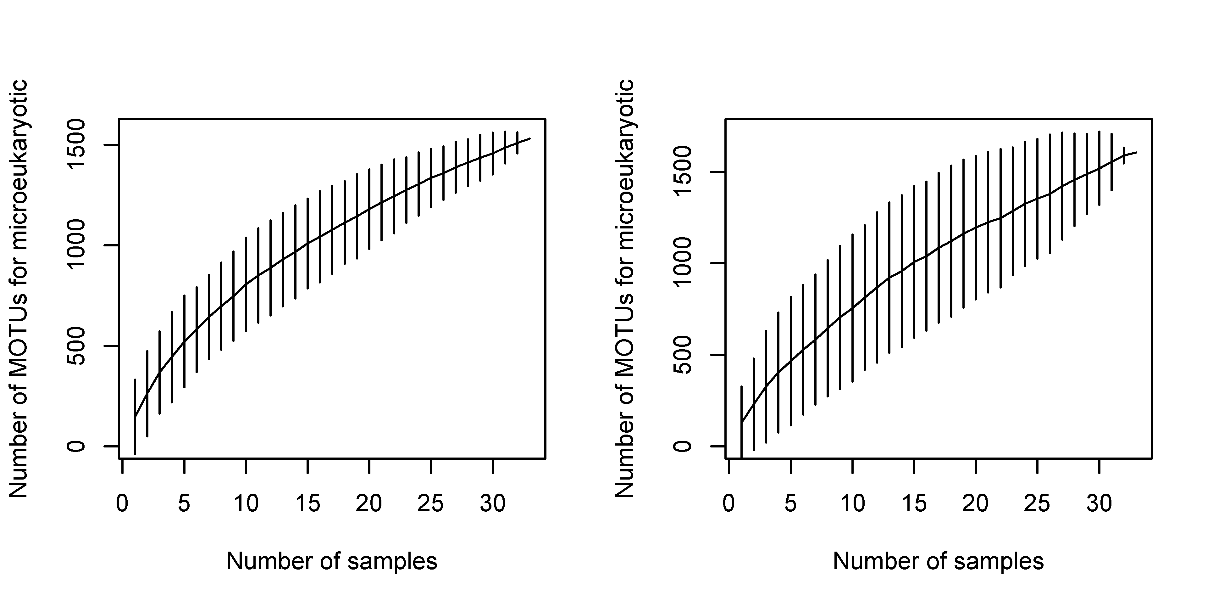


**Fig. S9** Boxplots for 5 environmental parameters in the Chacahua-Pastoria lagoon system.


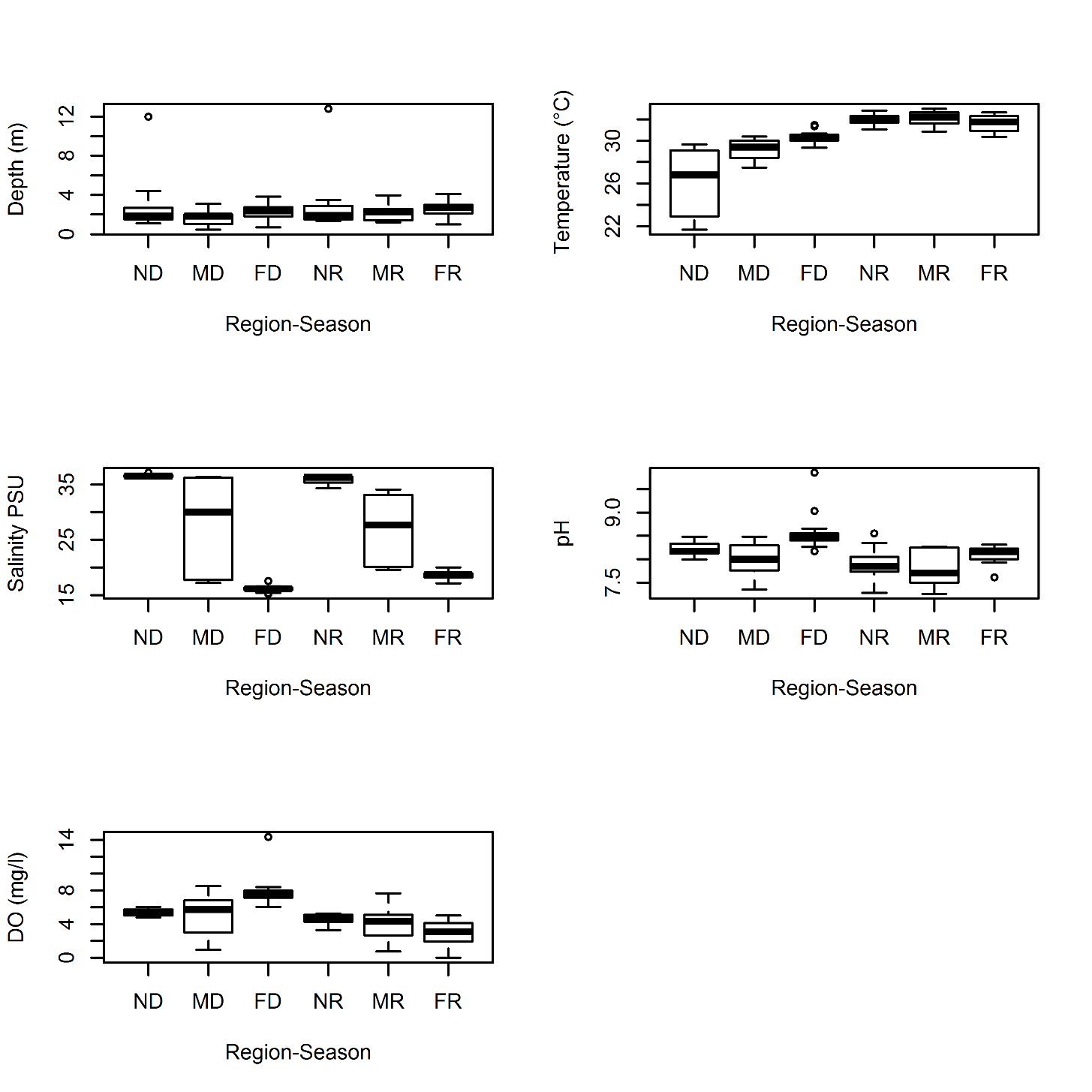


**Fig. S10** Boxplots for 5 environmental parameters in the Corralero-Alotengo lagoon system.


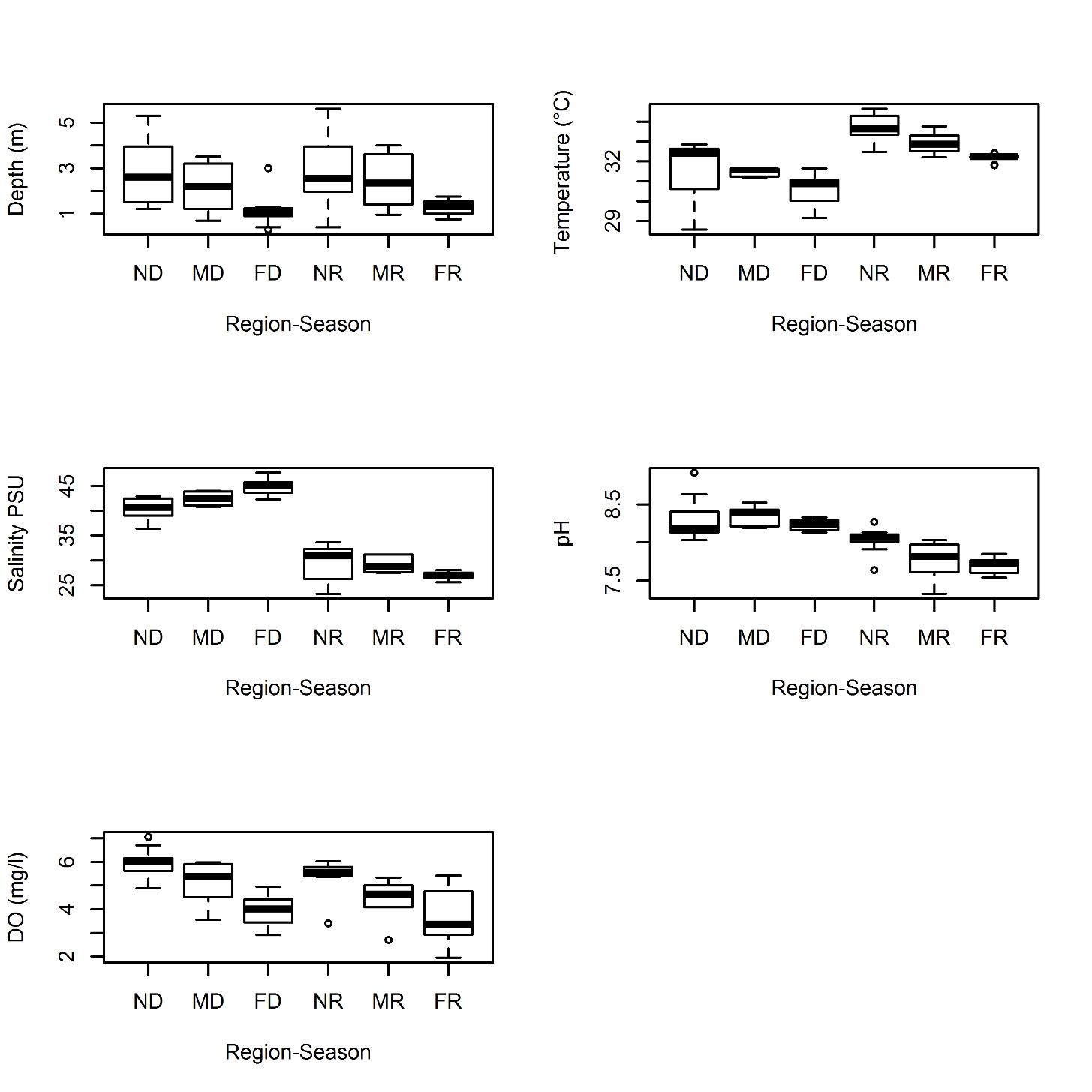


**Fig. S11** Pearson correlations for the richness (S) of metazoan MOTUs and the 5 environmental variables measured in the Chacahua-Pastoria lagoon system.


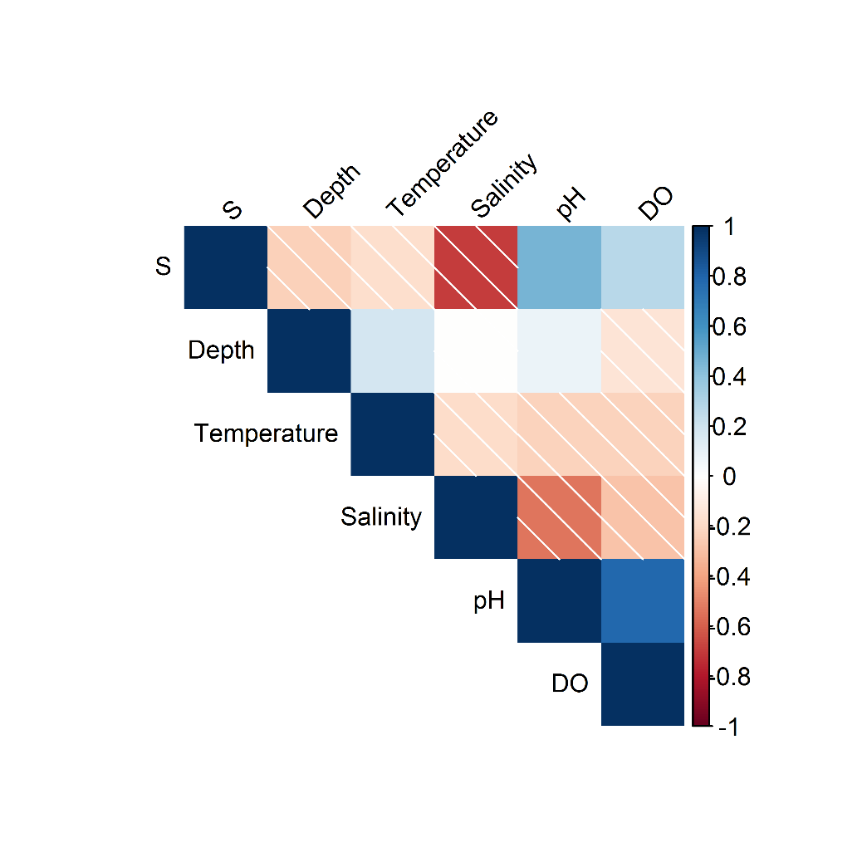


**Fig. S12** Pearson correlations for the richness (S) of metazoan MOTUs and the 5 environmental variables measured in the Corralero-Alotengo lagoon system


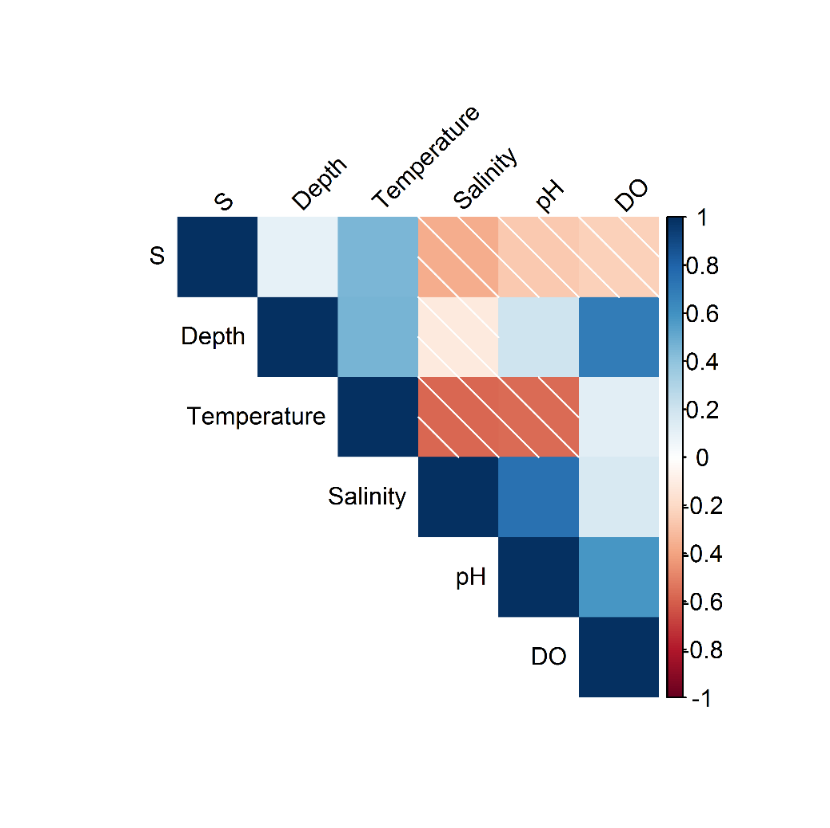


**Fig. S13** Pearson correlations for the richness (S) of micro-eukaryotic community MOTUs and the 5 environmental variables measured in the Chacahua-Pastoria lagoon system.


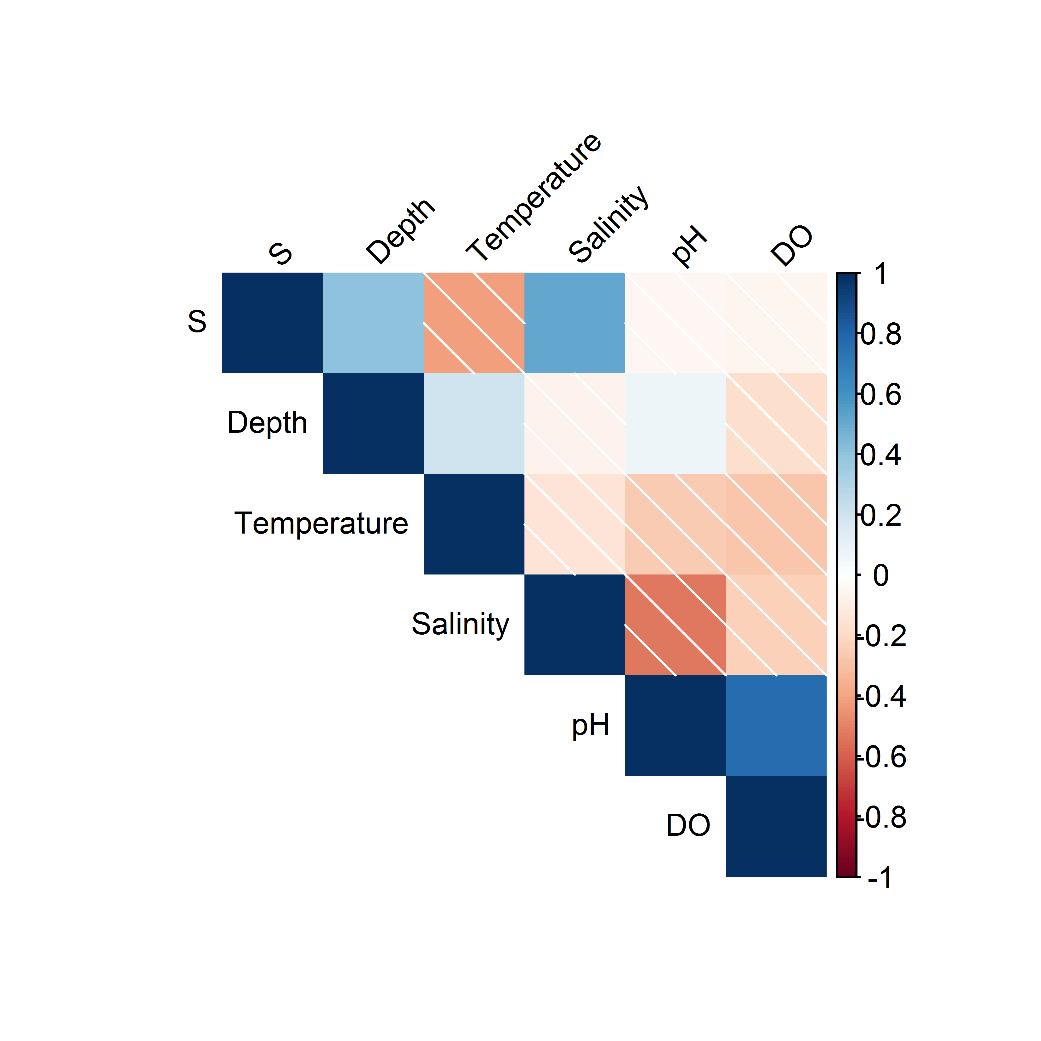


**Fig. S14** Pearson correlations for the richness (S) of micro-eukaryotic community MOTUs and the 5 environmental variables measured in the Corralero-Alotengo lagoon system.


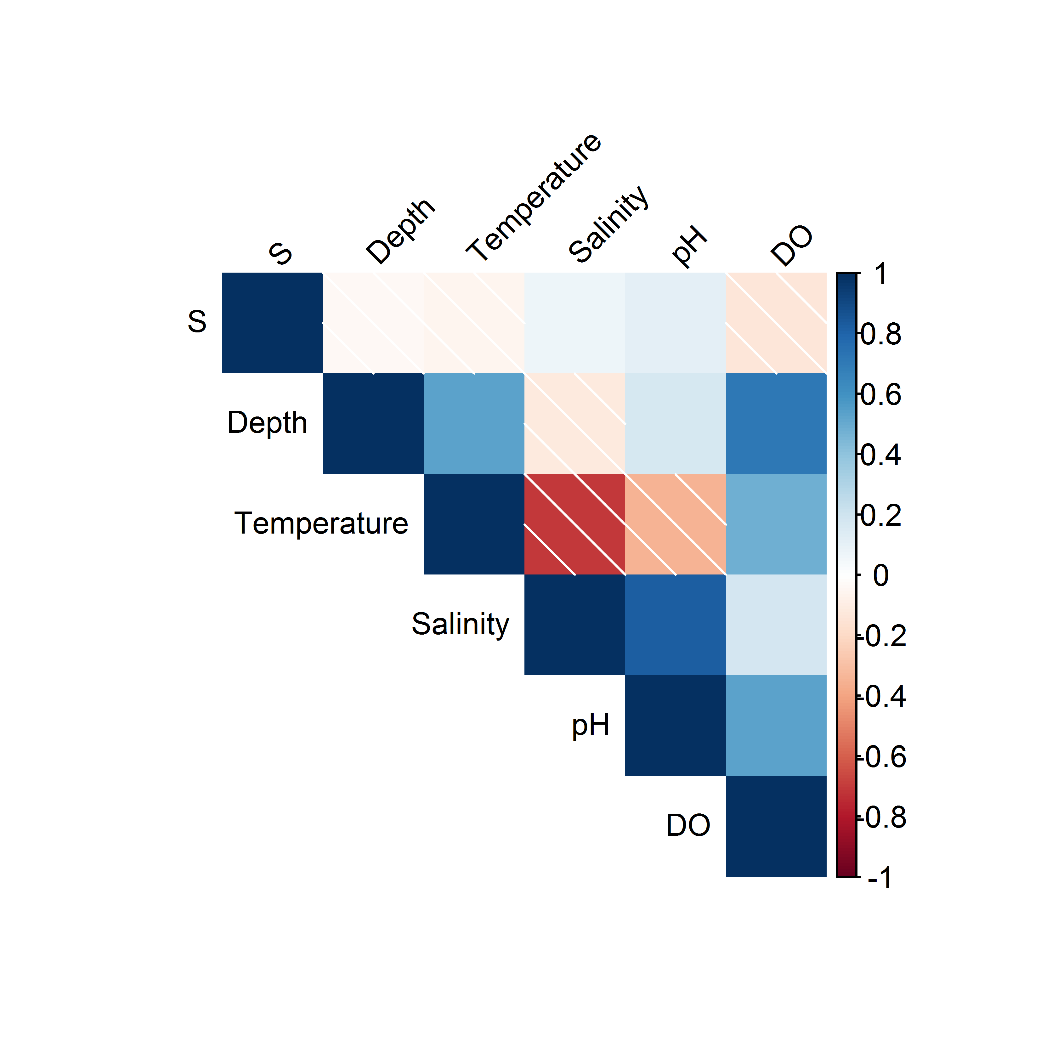


**Fig. S15** CCAs for metazoan and micro-eukaryotic communities identified in Corralero-Alotengo and Chacahua-Pastoria lagoon systems and their environmental characteristics (depth, salinity, temperature, pH, DO) during each season. Metazoans: A.) Corralero-Alotengo; CD: Corralero-Alotengo Dry season, CR: Corralero-Alotengo Rainy season. B) Chacahua-Pastoria; CHD: Chacahua-Pastoria Dry season, CHR: Chacahua-Pastoria Rainy season. Micro-eukaryotics: C.) Corralero-Alotengo; CD: Corralero-Alotengo Dry season, CR: Corralero-Alotengo Rainy season. D) Chacahua-Pastoria; CHD: Chacahua-Pastoria Dry season, CHR: Chacahua-Pastoria Rainy season. For all panels, the numbers in each sample indicate each of the different zones within the regions of the lagoon systems.


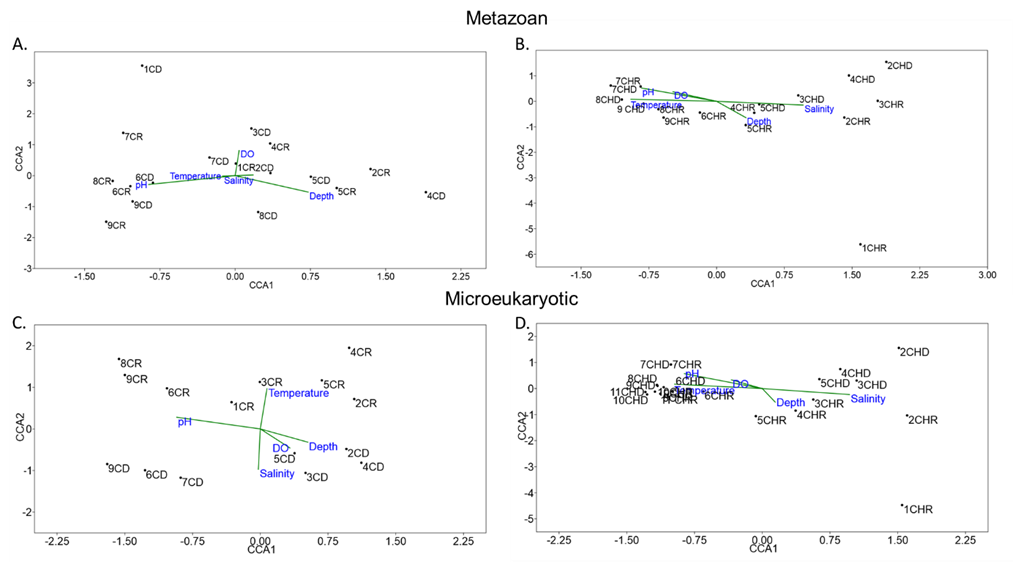

Supplement: Supplementary file 5 — Supplementary Information 5. [file 41598_2022_13653_MOESM5_ESM.docx]
